# Global and Local Deviance Effects in the Processing of Temporal Patterns

**DOI:** 10.1111/nyas.70173

**Published:** 2025-12-24

**Authors:** Dunia Giomo, Romain Brasselet, Gianfranco Fortunato, Domenica Bueti

**Affiliations:** ^1^ International School for Advanced Studies (SISSA) Trieste Italy; ^2^ Department of Human Neurosciences Sapienza University of Rome Rome Italy; ^3^ Sorbonne Université, INSERM, CNRS, Institut de La Vision Paris France

**Keywords:** finger tapping, local–global, rhythm, synchronization‐continuation, temporal regularity, temporal sequence

## Abstract

Perceptual and sensorimotor events are often experienced as temporal patterns, that is, identified as sequences based on their temporal features. While current timing models propose separate mechanisms supporting the processing of single intervals and temporal patterns, they leave partially unclear whether the latter entails the processing of both individual intervals and the overall structure of a pattern, or only one of these features. Here, we narrowed this question down by investigating how violations of regularity within the individual intervals of a temporal sequence (i.e., local violations) and in its overall structure (i.e., global violations) differentially affect its reproduction. We tested these violation effects in three experiments in which the sequences were experienced either in the visual or auditory domain and had either simple or complex structures. Results showed that the precision in reproducing simple visual and auditory patterns was primarily affected by local violations, whereas global violations mostly impacted the reproduction of visual patterns with complex structures. These detrimental effects were partially explained by rescaling and bias effects in the reproduced patterns. Overall, our findings indicate that the processing and reproduction of temporal patterns differentially weigh individual intervals and global structure, depending on sensory modality and, for visual patterns, on structural complexity.

## Abstract 2

Our experience of the world is inherently structured by temporal patterns. Yet a full understanding of how we process such patterns is still lacking. Across three finger‐tapping experiments employing the local–global paradigm, we show that the processing and reproduction of temporal patterns rely on a differential weighting of their individual elements and overall structure. This weighting depends on both the structural complexity of the patterns and the sensory modality in which they are experienced.

## 1 Introduction

Experiencing an awkwardly long moment of silence during a conversation or distinguishing an “SMS” (… ‐‐ …) from an “SOS” (… ‐‐‐ …) message delivered in Morse code requires two very different types of temporal computations that the human brain is equally able to perform efficiently and effortlessly. In the first case, evaluating awkwardness requires estimating the duration of a single event (the moment of silence), whereas in the second, the brain must compare multiple durations across two temporal patterns and assess their relationships. The ability to process and represent such different forms of temporal information is critical for a wide range of sensory and motor experiences.

Although the underlying mechanisms and neural underpinnings are still a matter of investigation and debate in timing literature, it is generally acknowledged that for the human brain, the processing of a single duration is a different operation than the processing of a temporal pattern (for a review, see Ref. [1]). In line with this distinction, a longstanding model in timing research proposes two distinct mechanisms for the processing of the absolute duration of events (*interval based*) and the processing of their relative duration (*beat based*) [2, 3, 4, 5, 6, 7, 8]. Although this model can account for a wide range of temporal judgments and types of temporal expectations, the experimental paradigms used to test it tend to emphasize a dichotomy between timing strategies (see, for instance, Refs. [9, 10, 11]), and they do not allow to observe whether and how these two strategies may both contribute to the processing of temporal patterns. As a result, these experimental paradigms do not adequately address whether and how humans encode and represent all the features of a temporal pattern—such as individual durations, their order, and the overarching structure. This leaves our understanding partially unresolved to whether temporal pattern processing involves additive processing of individual elements, holistic structural representation, or both.

A potentially useful approach to address this general question comes from several studies on sequence processing, particularly those investigating how the brain processes the regularity of sequences at different hierarchical levels and how it can build predictions on each level of regularity [12]. These studies usually fall within the general framework of predictive coding models [13] and their focus is on the neural responses associated with the recognition or violation of these regularities (for a review, see Ref. [14]). The experimental paradigms employed in these studies enable the simultaneous investigation of multiple hierarchical features of a sequence, as they are specifically designed to elicit responses to violations at each hierarchical level. As such, these paradigms serve as valuable tools to examine whether and how humans encode and represent multiple features of a temporal pattern, such as individual durations and overall structure.

In the present study, we adopted this approach to investigate how the reproduction of a temporal sequence is affected by regularity violations of its individual elements and/or of its overall structure. To operationalize this question, we adapted the local–global paradigm [15] to the temporal domain. This paradigm was originally developed to differentiate neurophysiological signatures associated with conscious and automatic processing of different levels of regularity in a sequence [16, 17]. Specifically, this paradigm allows to orthogonally manipulate violations within the single elements of a sequence (local violations) and in its overall structure (global violations). In relation to our research question, the extent to which local and global violations affect the reproduction of a temporal sequence, and produce distinct behavioral signatures, serves as a proxy for the relative weighing of individual elements and overall structure in temporal sequence processing. Specifically, if violations within the sequence's basic elements affect its reproduction, this would suggest that individual elements play a crucial role in the processing of the sequence. In this case, we would expect a drop in the precision of single taps whenever a local violation occurs, similar to what is observed in phase perturbation paradigms in sensorimotor synchronization [18]. Likewise, though not exclusively, if violations of the sequence's overall structure also lead to distortions in reproduction, we would expect a decrease in tapping precision across all intervals of any globally deviant sequence. This would support the notion that this higher‐level regularity is also encoded and represented. Conversely, if only one level of violation influences reproduction, this would indicate a more exclusive, feature‐specific processing strategy. Thus, our approach, by producing feature‐specific violation signatures, can potentially offer an alternative route for investigating temporal pattern processing, possibly complementing more traditional experimental approaches specifically focused on differentiating beat‐ versus interval‐based types of processing.

We conducted three behavioral experiments to investigate the effects of local and global violations of regularity on the reproduction of temporal sequences, and additionally, whether these effects would change if the sequences were experienced in the visual or auditory domain, and whether their structure was simple or complex. In all experiments, we manipulated the temporal features of the sequences (instead of their pitch as in the original paradigm [15]) and asked our participants to reproduce them in a Synchronization‐Continuation task [19, 20]. In Experiment 1 (Exp1V), we employed simple sequences made of empty intervals (i.e., silent gaps) marked by visual stimuli. In Experiment 2 (Exp2A), we used the auditory version of the sequences used in Exp1V. In Experiment 3 (Exp3VC), we used complex sequences marked by visual stimuli.

The key feature of the local–global paradigm is its incorporation of hierarchical violations within a sequence. In the current study, our primary goal was to examine how each sequence was reproduced based on its local–global status. To this end, we adopted a data analysis approach that simultaneously captures the reproduction of individual elements and the overall sequence structure, combining signal processing (frequency‐domain) techniques with shape‐mapping (time‐domain) methods.

## 2 Methods

### 2.1 Participants

#### 2.1.1 Exp1V

Thirty participants (9 males; 2 left‐handed; mean age 25 ± 5.1; age range 19−45) were recruited. Eleven of them reported different levels of musical expertise (5 amateur musicians, mean = 6.2 years; 6 with formal music/dance training, mean = 7.8 years).

#### 2.1.2 Exp2A

Twenty‐nine participants (11 males; 3 left‐handed; mean age 23 ± 3; age range 19−32) were recruited. The sample included 10 amateur musicians (mean = 5.2 years) and 8 people with formal training (mean = 8.4 years).

#### 2.1.3 Exp3VC

Twenty‐nine participants (10 males; 2 left‐handed; mean age 23.4 ± 3.5; age range 18−35) were recruited. The sample included six amateur musicians (mean = 4.2 years) and seven people with formal training (mean = 7.4 years).

All participants gave written informed consent before starting the experiment. The experimental protocols were approved by the SISSA Ethics Committee (n. 11175‐III/13) and complied with the Declaration of Helsinki. All participants were naive to the purposes of the study and received fixed monetary compensation after completing the task. Four participants were recruited for two out of three experiments (two for Exp1V and Exp2A; one for Exp1V and Exp3VC; one for Exp2A and Exp3VC), with several weeks between studies. They were debriefed only at the end of the data collection. The final samples used for the analysis (see Section 2.5.1 for details on exclusion criteria) were *n* = 26 for Exp1V (8 males; 2 left‐handed; mean age 24.6 ± 5.3; age range 19−45), *n* = 27 for Exp2A (10 males; 2 left‐handed; mean age 23 ± 3.1; age range 19−32), and *n* = 26 for Exp3VC (10 males; 2 left‐handed; mean age 22.7 ± 2.7; age range 18−27). None of the nine excluded participants had any musical expertise.

### 2.2 The Local–Global Paradigm

**FIGURE 1:**
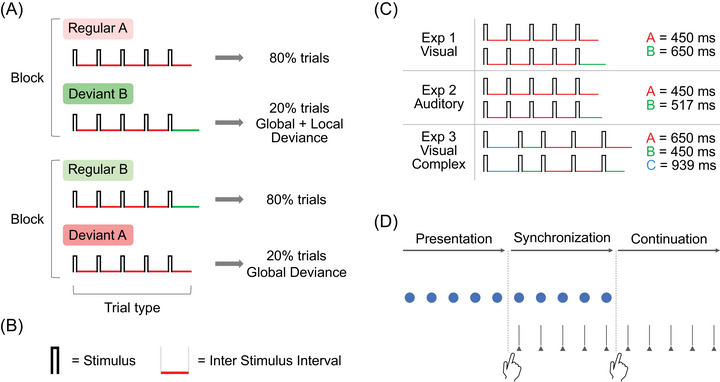
Local–global paradigm and task structure. (A) In one block type, regular trials contain the locally regular sequence AAAAA (shaded in light red), and deviant trials contain the locally deviant sequence AAAAB (shaded in green): in this condition, deviant trials contain a deviance both at the local and global level. In the other block type, regular trials contain the locally deviant sequence AAAAB (shaded in light green), and deviant trials contain the locally regular sequence AAAAA (shaded in red): in this condition, deviant trials contain a deviance only at the global level. (B) The temporal sequences used in all experiments were made of empty intervals (Inter Stimulus Interval) marked by brief visual or auditory markers (Stimulus). (C) Sequences of empty intervals used in each experiment. (D) Synchronization‐Continuation task. Each trial of the task has three phases: at first, the participant simply attends to a rhythm given by an external stimulus (Presentation phase), then has to tap the finger in synchrony with the rhythm (Synchronization phase), and at last, continues to tap the rhythm without any external reference (Continuation phase).

The local–global paradigm [15] employs two types of sequences made of basic elements (here, temporal intervals) A and B: the first is composed of five identical elements (AAAAA) and the second is made of four identical elements plus a fifth different one (AAAAB). Sequence AAAAA is defined as “locally regular,” given the absence of variation within its elements, whereas sequence AAAAB is defined as “locally deviant” because it comes with an intrinsic regularity violation within its elements. Each trial consists of a few repetitions of only one type of 5‐element sequence (e.g., AAAAB AAAAB AAAAB…). In each experimental block, the relative frequency of the trials, containing either one sequence or the other, determines which sequence is identified as “globally regular” and which as “globally deviant.” Specifically, in one experimental condition (block type), the locally regular sequence AAAAA makes up 80% of the trials, making it the globally regular sequence, while the locally deviant sequence AAAAB makes up the remaining 20% of the trials, thus serving as the globally deviant sequence (Figure 1A).

In the other condition, this frequency distribution is reversed: the sequence AAAAB becomes the globally regular sequence (80% of the trials), and the sequence AAAAA becomes the globally deviant sequence (20% of the trials). This orthogonal manipulation results in two violation conditions: in the first block type, the regularity violation is both at the local and global level, that is, both within the elements and in the overall structure of the sequence. In the second block type, the regularity violation is only at the global level, that is, the change is only in the overall structure of the sequence. In the current study, we adapted this paradigm to the temporal domain, using sequences made of empty intervals (i.e., silent gaps) interleaved by brief visual or auditory markers (Figure 1B); hence, the crucial changing feature was the empty temporal interval between either visual or auditory markers. In Exp1V and Exp2A (Figure 1C), we used the same sequence structures used in the original paradigm (AAAAA and AAAAB), whereas for Exp3VC, we used two complex sequences (i.e., neither isochronous nor pseudo‐isochronous), while maintaining the local regularity/deviance contraposition between the fourth and the fifth element (CBAAA and CBAAB).

### 2.3 Stimuli and Apparatus

#### 2.3.1 Stimuli

In the visual experiments Exp1V and Exp3VC, the sequences were made of empty intervals marked by brief blue disks (33 ms, radius 32 pixels) appearing at the center of a computer screen (monitor BenQ‐XL2720Z, 35 × 28 cm, resolution 1920 × 1080 pixels). In the auditory experiment Exp2A, the empty intervals were marked by beeps (33 ms pure tones of 1000 Hz, 2‐ms rise and fall time filter) delivered through noise‐cancelling headphones. Both types of markers were created in MATLAB (R2019b, The MathWorks, Inc.).

#### 2.3.2 Apparatus

In all experiments, we employed a custom‐made set‐up (www.cynexo.com) that allowed a single common recording system and timescale for collecting participants’ responses and keeping track of each stimuli‐related event. The setup included a microcontroller Arduino Due (www.arduino.cc) connected to a response pad and a photodiode attached to the monitor. The photodiode served as a relay between monitor and microcontroller, allowing precise recording of the onset of sequence markers (see below). In Exp2A, this apparatus also served to deliver auditory stimuli by integrating a second microcontroller (Arduino Uno) equipped with an Adafruit Wave Shield (www.adafruit.com), to which headphones were directly connected.

Each trial series was generated using MATLAB and Psychtoolbox [21, 22, 23], where each stimulus onset (i.e., the onset of each marker of the sequences) was coded as a visual trigger appearing on one corner of the screen (hidden from participants). Each trigger onset was recorded by the microcontroller, via a simultaneous change in the photodiode status. In Exp1V and Exp3VC, each visual trigger was paired with the simultaneous presentation at the center of the screen of each visual marker. In Exp2A, the visual trigger sequentially prompted the two microcontrollers to play a beep, marking each auditory event onset. Technical tests showed a constant delay of 20 ms in sound delivery from the visual trigger onset to the headphones, with a negligible standard deviation (in the microsecond scale). The response pad was equipped with a plastic button, where participants were instructed to tap, and sent a binary signal (1 if the button was pressed, otherwise 0) to the microcontroller at 500 Hz sampling rate. To reach high temporal precision and avoid delay, the microcontroller was programmed to asynchronously read the response event buffer via an interrupt service routine. To minimize any mechanical delay, the plastic button was designed to have a short stroke. To avoid any auditory feedback produced by the button press, in Exp1V and Exp3VC, white noise was delivered through headphones. In Exp2A, instead, participants wore noise‐canceling headphones. In Exp1V and Exp3VC, participants’ gaze was tracked using an EyeLink 1000 device (SR Research) to ensure that visual fixation was maintained at the center of the screen.

### 2.4 Procedure and Task

Participants sat comfortably in front of the computer screen in a dimly lit room. After receiving general instructions on the experimental procedure, they were asked to wear headphones, adjust the sound volume to a comfortable level, and place their head on a chin rest. In the main experiment, participants performed three blocks per experimental condition, six in total. Blocks of the same experimental condition were performed consecutively. The condition order was counterbalanced across subjects. The structure of each block was the following: to establish the condition‐specific global regularity, the first five trials (“familiarization” trials) contained only the globally regular sequence; familiarization trials were followed by 10 experimental trials, eight of which contained the globally regular sequence and two the globally deviant. Within a block, the order of the experimental trials was randomized, thus any of them could contain the globally deviant sequence. In total, for each experimental condition, participants performed 24 trials containing the globally regular sequence and six trials containing the globally deviant one. No feedback was given during the main experiment. Participants were allowed to take short breaks between blocks.

Participants performed a brief training before starting the main experiment. The training was a simplified version of the main experiment, keeping the same block structure described above. It consisted of two blocks, one per condition, each made of two familiarization trials followed by five experimental trials (four containing the globally regular sequence and one the globally deviant). A feedback message on tapping accuracy was shown on the screen at the end of each training trial.

#### 2.4.1 Sequences’ Structure

For Exp1V (Figure 1C), the empty intervals composing the sequences AAAAA and AAAAB were set to A = 450 ms and B = 650 ms, with B 45% longer than A. This difference was chosen to ensure sufficient discriminability between the intervals. For Exp2A, the interval B was reduced to 517 ms (15% longer than A) to take into account the finer temporal sensitivity of the auditory system [24] and maintain a comparable level of difficulty between auditory and visual tapping tasks [25]. For Exp3VC, the empty intervals composing the sequences CBAAA and CBAAB were set to A = 650 ms, B = 450 ms, and C = 939 ms, with each interval being approximately 45% longer than the one immediately shorter. In this case, the shortest interval also served as the locally deviant element of the sequence. This choice allowed us to control for potential confounding effects in Exp1V and Exp2A, where interval B was longer than A, thus ensuring that any observed effects were due to its status as a locally deviant rather than its absolute length. To avoid any transfer of temporal learning to the main experiment, the sequences used in the training consisted of different intervals (A = 500 ms, B = 700 ms in Exp1V; A = 500 ms and B = 575 ms in Exp2A; A = 700 ms, B = 500 ms, and C = 980 ms in Exp3VC).

#### 2.4.2 Synchronization‐Continuation Task

Each trial consisted of three phases (Figure 1D). *Presentation phase*: After a brief interval (randomly selected between 0.9 and 1.7 s), the sequence was presented five times to the participants, who simply had to pay attention to it. *Synchronization phase*: Participants were prompted by an on‐screen message to begin tapping on the response pad in synchrony with the external stimulus (either visual or auditory markers), for five sequence repetitions. *Continuation phase*: Once the external stimulus disappeared, participants were required to continue tapping at the same pace, repeating the sequence just presented. A message on the screen signaled the end of the trial, which occurred after approximately three sequence repetitions plus an interval randomly selected between 0.9 and 1.7 s. The start of the next trial was self‐paced. During the Presentation and Synchronization phases, each sequence repetition followed the previous one immediately, with the sixth marker of one sequence serving as the first marker of the next. This continuous stream of stimuli was designed to avoid introducing additional temporal manipulations (e.g., an inter‐sequence interval) that could have biased the way participants grouped the elements of the sequences.

### 2.5 Data Analysis

#### 2.5.1 Preprocessing

All preprocessing steps to extract event onsets were performed trial‐wise for each participant, using MATLAB custom codes. Stimuli and tapping signals were recorded by our apparatus as time series of 0 and 1, where 1 represents an event onset. Specifically, each stimulus onset corresponded to a change in the photodiode status prompted by the appearance of the visual trigger on the screen, while each tap onset corresponded to a button press. For both time series, each positive derivative was identified as an onset and stored with its timestamp. For Exp2A, the actual timing of each beep onset was calculated by adding a constant delay of 20 ms (see *Apparatus*). Tap onsets following a previous one within a window of 250 ms were considered either as artifacts or “false” onsets (e.g., double tap), hence discarded. Isolated tap onsets detected during the Presentation phase or at the end of the Continuation phase (>2 s before/after the next/previous onset) were also excluded. All familiarization trials were excluded from the analysis. Nine participants (four in Exp1V, two in Exp2A, and three in Exp3VC) were excluded due to their poor performance, such as inability to synchronize with the external stimulus or the presence of long gaps in the tapping in more than 25% of trials. The final sample sizes used for the analysis were *n* = 26 for Exp1V, *n* = 27 for Exp2A, and *n* = 26 for Exp3VC.

#### 2.5.2 Frequency‐Domain Analysis

To explore the effects of our experimental manipulations on the precision of tapping performance, we analyzed tapping data in the frequency domain, by computing and comparing the power spectra of stimuli sequences and of participants’ performance.

**FIGURE 2:**
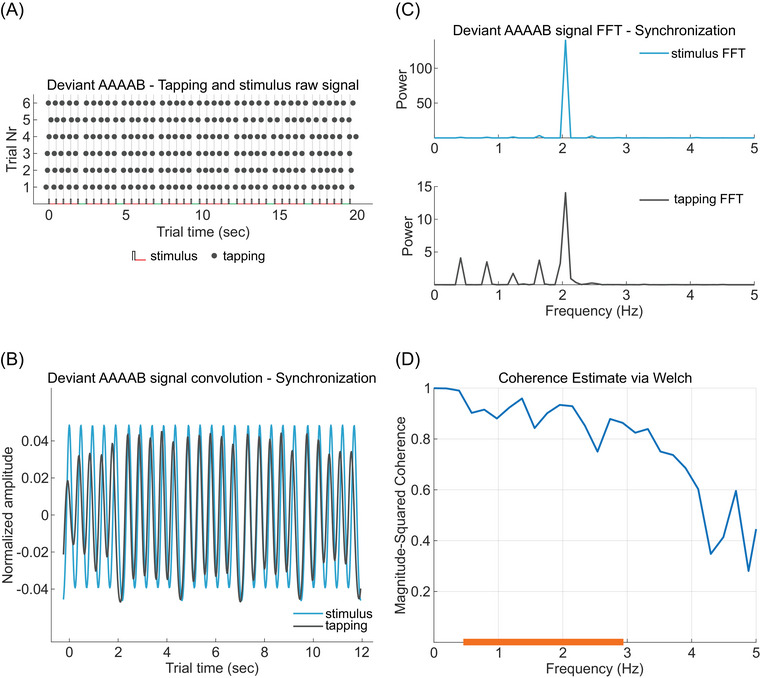
Stimuli and tapping signal convolution and magnitude squared coherence (MSC) analysis pipeline (data from one participant in the condition *globally deviant AAAAB* in Exp2A). (A) Stimuli series and participant's raw tapping performance. For each trial, tapping onsets (dark gray dots) are plotted time‐aligned to the stimuli sequences (alternating black vertical and red/green horizontal lines, representing stimulus onset and inter‐stimulus intervals, respectively, as in Figure 1B). (B) Convolution of tapping (dark gray) and stimulus (blue) signals from the Synchronization phase of the same participant. (C) Power spectrum of stimulus and tapping signals for the same participant, color coded as in (B). (D) MSC values obtained from the comparison between stimuli and tapping power spectra, plotted as a function of frequency. The orange bar on the x‐axis highlights the frequency range that includes the power spectrum of the sequence AAAAB (approximately between 0.5 and 3 Hz). Statistical analysis on MSC values focused within this range. Abbreviation: FFT, fast Fourier transform.

For each trial, all stimuli and tap onsets were recoded with respect to the first stimulus onset of the Synchronization phase (Figure 2A), discarding all events detected earlier than 250 ms before this time point. The resulting time series from each combination of trial type X sequence type (globally regular AAAAA, globally deviant AAAAB, globally regular AAAAB, globally deviant AAAAA) were concatenated and convolved with a kernel density estimator (temporal resolution = 20 ms, bandwidth = 0.1), which transformed the discrete event series into a continuous probability density function of event onsets in time (an example of convolved stimulus and tapping signals is presented in Figure 2B). This procedure led to four convolved tapping signals and four convolved stimulus signals per participant. Each signal was split into a Synchronization and a Continuation series. The cutting point was the end cycle of the last stimulus onset of the Synchronization phase. We then computed the fast Fourier transform of the convolved stimuli series to identify the frequency spectrum of the sequences used. The spectrum of sequence AAAAA, being isochronous, has a single power peak around 2 Hz, whereas the spectrum of AAAAB in Exp1V lies within 0.3 and 2.3 Hz with the greatest peak around 2 Hz. The spectrum of AAAAB in Exp2A is between 1.6 and 2.5 Hz, with a peak around 2 Hz (as shown in the upper panel of Figure 2C). For Exp3VC, both frequency spectra defining sequences CBAAA and CBAAB lie within 0.25 and 2.8 Hz.

For each participant, task phase, trial type, and sequence type, we compared the power spectra of the convolved tapping and stimulus signals using magnitude squared coherence (MSC). MSC was computed via Welch's overlapped averaged periodogram method [26], with a sampling rate of 50 Hz and 50% overlap between consecutive frequency segments. MSC is a standardized precision measure, ranging between 0 and 1, and it evaluates the similarity (i.e., coherence) between the power spectra of two signals, computed as the ratio between their cross‐power spectral density and auto‐spectral density functions [27], as follows:

$$C_{xy}\left(f\right)=\frac{{\left|G_{xy}\left(f\right)\right|}^{2}}{G_{xx}\left(f\right)\;G_{yy}\left(f\right)},$$

where *G_xy_(f)* represents the cross‐spectral density and *G_xx_(f)* and *G_yy_(f)* the auto‐spectral density, respectively. As MSC returns a value of coherence for each frequency bin in the spectrum (an example of MSC values across the full frequency spectrum is shown in Figure 2D), we focused our analysis only on the sequence‐specific frequency ranges listed above (highlighted in orange in Figure 2D) and calculated their average MSC value. We assessed the effects of trial type (globally regular vs. globally deviant), sequence type (locally regular vs. locally deviant), and task phase (Synchronization vs. Continuation) on average MSC values using a linear mixed effects (LME) model, with participants as the random intercept. MSC was computed in MATLAB. The LME model was fitted in R, using *lme4* [28] and *lmerTest* [29] packages, and the *MuMIn* package [30] was used for calculating *R* ^2^ statistics. Overall effects were tested using Type III ANOVA with Satterthwaite's approximation of degrees of freedom. Effect sizes were measured with partial eta squared calculated using the *effectsize* package [31]. Pairwise comparison tests were conducted using the *emmeans* package [32], applying Kenward−Roger's approximation of degrees of freedom and Tukey's method for *p*‐value adjustment. The alpha level was set to 0.05.

#### 2.5.3 Procrustes Analysis

To explore further the effects of our experimental manipulations on participants’ performance, independently of precision measures, we carried out a second analysis focused on the internal consistency of tapping signals. Specifically, we employed a Procrustes analysis [33], a mapping technique that applies a Euclidean transformation to find the best match between two shapes or linear signals by simultaneously considering different nonrandom sources of distortions in the to‐be‐matched signal. In our tapping signals, these distortions are quantified through two components, namely, bias and scaling (see Figure S1). In tapping behavior, these components translate, respectively, into a constant anticipation or delay of tap onset with respect to stimuli onset (*bias*, with negative values expressing an anticipation and positive values a delay), and a slowed down/speeded up reproduction of the stimuli series (*scaling*, with values above 1 indicating acceleration and below 1 deceleration). Once these components are taken into account, the remaining distortions in the transformed signal are considered more noise‐related.

To apply the Procrustes algorithm, in all three experiments, additional preprocessing steps were performed to obtain tapping and stimuli signals of matching length (for details, see Method S1). Each matched tap‐stimulus time series was further split into its Synchronization and Continuation parts, based on the onset timing of the first stimulus of the Continuation phase, and separately transformed by the Procrustes algorithm. This led to two biases and two scaling values for every trial (one per task phase). We assessed the effects of trial type (globally regular vs. globally deviant) and sequence type (locally regular vs. locally deviant) on bias and scaling values using separate LME models for Synchronization and Continuation phases, with participants as random intercept (see Section 2.5.2 for software details).

## 3 Results

### 3.1 Magnitude Squared Coherence

We analyzed tapping signals in the frequency‐domain using a measure of precision of performance, that is, MSC, which expresses the degree of similarity/coherence between the tapping and stimulus power spectra. For each experiment, we fitted an LME model on mean MSC values to assess the effects of trial type, sequence type, and task phase (model formula: MSC ∼ trial type * sequence type * task phase + (1 | subject)).

#### 3.1.1 Exp1V

**FIGURE 3:**
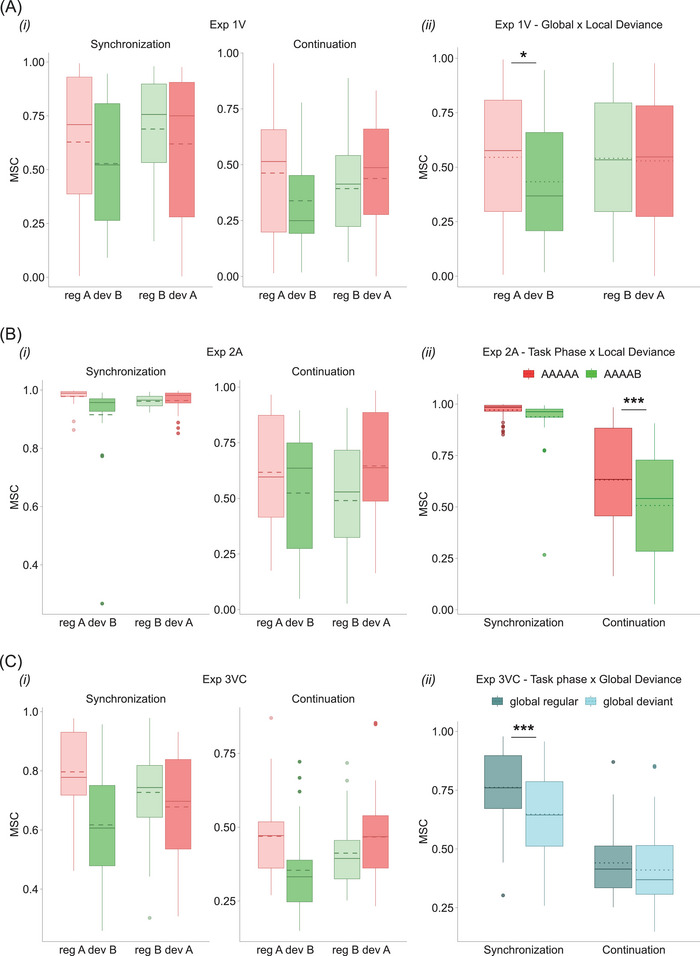
Magnitude squared coherence (MSC) analysis. (A) Distribution of MSC values for Exp1V (*i*) grouped by task phase, trial type, and sequence type (color‐code: light red = globally regular A; green = globally deviant B; light green = globally regular B; red = globally deviant A). In Exp1V, a significant drop in MSC values was observed only for the locally and globally deviant sequence AAAAB, as revealed by pairwise comparison tests and shown in the interaction plot in (A*ii*). (B) Distribution of MSC values for Exp2A (*i*) grouped and color‐coded as in (A). MSC analysis for Exp2A showed a generalized detrimental effect of local deviance, independent of the global status of the sequence. This local deviance effect was most strongly present during the Continuation phase, as shown in the interaction plot in (B*ii*). (C) Distribution of MSC values for Exp3VC (*i*) grouped and color‐coded as in (A). MSC analysis highlighted both local deviance and global deviance effects for complex visual sequences (Exp3VC). In particular, the global deviance effect was significantly present during the Synchronization phase irrespective of the local status of the sequence, as shown in the interaction plot in (C*ii*). Boxplots’ borders represent interquartile range. Solid lines and dotted lines represent median and mean MSC values, respectively. *p < 0.05; ***p < 0.001.

For the first experiment (Figure 3A), the ANOVA on the LME model estimate (*R* ^2^_marg_ = 0.153, *R* ^2^_cond_ = 0.63) showed significant main effects of trial type (MSC_globReg_ = 0.543, MSC_globDev_ = 0.481, *F*(1, 175) = 6.05, *p* = 0.015, $\eta_{p}^{2}$ = 0.03); of sequence type (MSC_A_ = 0.537, MSC_B_ = 0.487, *F*(1,175) = 3.94, *p* = 0.049, $\eta_{p}^{2}$ = 0.02); and of task phase (MSC_cont_ = 0.408, MSC_synch_ = 0.616, *F*(1, 175) = 68.02, *p* < 0.0001, $\eta_{p}^{2}$ = 0.28). We also found a potential, albeit nonsignificant, interaction between trial and sequence type (*F*(1, 175) = 3.26, *p* = 0.073, $\eta_{p}^{2}$ = 0.02). No other interaction was significant (all effects reported in Table S1). Pairwise comparison tests on the interaction between trial and sequence type showed a significant difference (Figure 3A *ii*) between the globally deviant sequence AAAAB and all the other sequences (GlobaReg_A_—GlobDev_B_ = 0.11, *t*(175) = 3.14, *p* = 0.01; GlobDev_B_—GlobReg_B_ = −0.11, *t*(175) = −3.01, *p* = 0.015; GlobDev_A_—GlobDev_B_ = 0.10, *t*(175) = 2.68, *p* = 0.04; all contrasts reported in Table S4). These last results indicate that the globally and locally deviant AAAAB had a lower MSC than all three other sequences. Considering the block structure of our experiments, the results of Exp1V overall revealed that the passage from the globally regular AAAAA to the globally deviant AAAAB, that is, when global and local deviance are combined, had detrimental effects on performance, irrespective of the task phase (Figure S2A). On the contrary, in the other experimental condition, that is, where the passage is from the globally regular AAAAB to the globally deviant AAAAA, we found no such effect. Additionally, a general decrease of tapping precision characterized the Continuation phase, compared to the Synchronization, independent from the type of sequence reproduced.

#### 3.1.2 Exp2A

Concerning the auditory experiment (Figure 3B), the ANOVA on the LME model estimate (*R* ^2^_marg_ = 0.519, *R* ^2^_cond_ = 0.635) showed significant main effects of sequence type (MSC_A_ = 0.801, MSC_B_ = 0.723, *F*(1,182) = 11.94, *p* = 0.0007, $\eta_{p}^{2}$ = 0.06) and of task phase (MSC_cont_ = 0.569, MSC_synch_ = 0.955, *F*(1, 182) = 287.76, *p* < 0.0001, $\eta_{p}^{2}$ = 0.61). Results also showed a significant interaction between sequence type and task phase (*F*(1, 182) = 4.10, *p* = 0.044, $\eta_{p}^{2}$ = 0.02), since MSC values for the locally regular sequence AAAAA were significantly higher than those for the locally deviant AAAAB in the Continuation but not in the Synchronization phase (Figure 3B *ii*), as revealed by pairwise comparison tests (A_cont_−B_cont_ = 0.12, *t*(182) = 3.87, *p* = 0.0008; all contrasts reported in Table S5). No other main effects or interactions were significant (all effects reported in Table S2). As in Exp1V, we found the expected worsening of tapping performance during the Continuation phase, compared to the Synchronization. What is peculiar in Exp2A is the clear presence of a pure local deviance effect when using auditory patterns, meaning that the locally deviant AAAAB was always less precisely performed, independently of its global status (Figure S2B). This effect was evident during the Continuation phase, when the external reference was absent. However, potential differences between AAAAA and AAAAB during the Synchronization phase may have gone undetected due to near‐ceiling MSC values (i.e., approaching 1).

#### 3.1.3 Exp3VC

Regarding the study using complex visual sequences (Figures 3C and S2C), the ANOVA on the LME model estimate (*R* ^2^_marg_ = 0.475, *R* ^2^_cond_ = 0.729) showed significant main effects of trial type (MSC_globReg_ = 0.601, MSC_globDev_ = 0.529, *F*(1, 175) = 20.79, *p* < 0.0001, $\eta_{p}^{2}$ = 0.11); of sequence type (MSC_A_ = 0.603, MSC_B_ = 0.528, *F*(1, 175) = 22.41, *p* < 0.0001, $\eta_{p}^{2}$ = 0.11); and of task phase (MSC_cont_ = 0.425, MSC_synch_ = 0.705, *F*(1, 175) = 310.66, *p* < 0.0001, $\eta_{p}^{2}$ = 0.64). The latter effect, in line with Exp1V and Exp2A, reflects the expected worsening of performance in the Continuation phase. A significant interaction between trial type and task phase was found as well (*F*(1, 175) = 7.05, *p* = 0.0087, $\eta_{p}^{2}$ = 0.04; Figure 3C*ii*). Pairwise comparison tests revealed that this effect was driven by significantly lower MSC values for globally deviant compared to globally regular sequences during the Synchronization but not the Continuation phase (GlobDev_synch_—GlobReg_synch_ = −0.11, *t*(175) = −5.10, *p* < 0.0001; all contrasts reported in Table S6). No other interaction was significant (all effects reported in Table S3). Overall, results from Exp3VC showed that tapping precision was influenced by both local and global violations of sequence regularity. Specifically, locally deviant sequences were consistently associated with reduced precision, regardless of their global status. Additionally, globally deviant sequences, irrespective of their local status, were more difficult to synchronize with, as evidenced by the significantly lower MSC values for globally deviant, compared to globally regular, sequences during the Synchronization phase.

Taken together, the MSC analysis results indicate that with simple temporal patterns, as in Exp1V and Exp2A, local deviances significantly impair participants’ precision in reproducing a sequence. The local deviance effect appears to be greater when patterns are experienced in the auditory compared to the visual modality, as shown by the bigger effect size in Exp2A. Additionally, in the auditory domain, this effect appears to be totally independent from the familiarity with the overall structure of the pattern, that is, its global status. In contrast, in the visual modality, performance worsens when the local change also alters the overall structure of the sequence, indicating a combined global and local deviance effect. With more complex temporal patterns, as in Exp3VC, performance continues to be affected by local violations; however, synchronization accuracy is specifically impaired when the overall structure of the pattern is violated, reflecting a pure global deviance effect.

### 3.2 Procrustes Analysis

MSC is a static measure of spectral coherence, and it is sensitive to phase shifts and amplitude differences [34]. In other words, MSC cannot distinguish whether reduced tapping precision arises from a phase offset between the tapping and the stimulus, from spectral similarity occurring at slightly different frequencies, or from increased noise in the tapping signal. Behaviorally, this means that MSC does not differentiate whether performance deterioration is due to a consistent anticipation or delay in the tapping relative to the stimuli, or to a global acceleration or deceleration of the reproduced pattern. To quantify these components in tapping signals and separate them from pure noise, we explored the internal consistency of participants’ performances by means of a Procrustes analysis. For each participant, we derived bias and scaling values according to trial type, sequence type, and task phase. We fitted a separate LME model for the Synchronization and Continuation phases, using either the scaling or the bias as the dependent variable, with trial type and sequence type as fixed effects (model formula: scaling/bias ∼ trial type * sequence type + (1 | subject)).

#### 3.2.1 Exp1V

**FIGURE 4:**
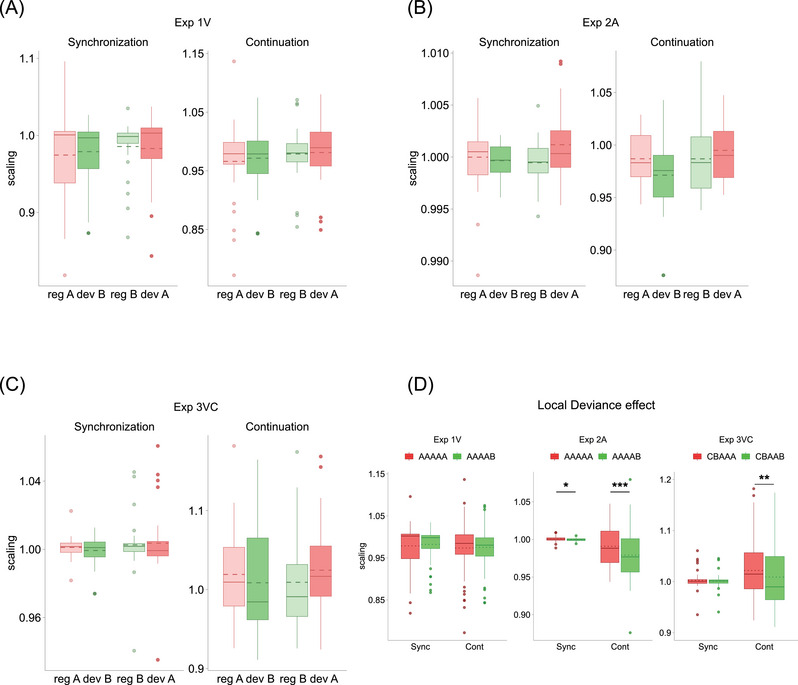
Procrustes analysis: scaling component. (A) Distribution of scaling values for Exp1V grouped by task phase, trial type, and sequence type (color‐code: light red = globally regular A; green = globally deviant B; light green = globally regular B; red = globally deviant A). In this experiment, only weak increases or decreases in scaling values were observed between each globally regular sequence and its respective globally deviant sequence, but none of these tendencies reached statistical significance. (B) Distribution of scaling values for Exp2A grouped and color‐coded as in (A). Statistical analysis revealed a general local deviance effect, as highlighted in (D). (C) Distribution of scaling values for Exp3VC grouped and color‐coded as in (A). For complex sequences, a statistically significant effect of local deviance was observed. (D) Local deviance effect on scaling values for Exp1V (left), Exp2A (center), and Exp3VC (right). In Exp2A and Exp3VC, scaling values differed significantly between locally regular and locally deviant sequences, whereas in Exp1V, they did not. However, scaling differences in Exp2A and Exp3VC translated in different tapping behaviors: in Exp2A, the tapping of the locally deviant AAAAB is slowed down compared to the baseline (scaling values < 1) and to the locally regular AAAAA, whereas in Exp3VC, the locally regular CBAAA is speeded up compared to baseline (scaling values > 1) and to the locally deviant CBAAB. Boxplots’ borders represent interquartile range. Solid lines and dotted lines represent median and mean scaling values, respectively. *p < 0.05; **p < 0.01; ***p < 0.001.

Concerning the scaling component in the first experiment, the ANOVA on the two LME models’ estimates (scaling‐synch: *R* ^2^_marg_ = 0.008, *R* ^2^_cond_ = 0.83; scaling‐cont: *R* ^2^_marg_ = 0.01, *R* ^2^_cond_ = 0.837) revealed only a significant interaction between trial type and sequence type in both task phases (scaling‐synch: *F*(1, 75) = 4.16, *p* = 0.045, $\eta_{p}^{2}$ = 0.05; scaling‐cont: *F*(1, 75) = 5.46, *p* = 0.022, $\eta_{p}^{2}$ = 0.07; all effects reported in Table S7). This effect was driven by differential changes, either mild decreases or increases, between each globally regular sequence and its corresponding globally deviant sequence (Figure 4A). However, this effect appeared not to be consistent enough, as all pairwise comparison tests failed to reach significance (all contrasts and means reported in Tables S10 and S11). Concerning the bias component (bias‐synch: *R* ^2^_marg_ = 0.006, *R* ^2^_cond_ = 0.83; bias‐cont: *R* ^2^_marg_ = 0.01, *R* ^2^_cond_ = 0.81), the ANOVA on the LME models’ estimates showed similar results (Figure S4A): only a significant effect for the interaction between trial type and sequence type in the Continuation model (bias‐cont: *F*(1, 75) = 4.74, *p* = 0.033, $\eta_{p}^{2}$ = 0.06), which did not reach statistical significance in the Synchronization model (bias‐synch: *F*(1, 75) = 3.59, *p* = 0.062, $\eta_{p}^{2}$ = 0.04; all effects reported in Table S13). However, pairwise comparison tests for bias values in the Continuation model failed to reach significance too (all contrasts and means reported in Table S16). Although not fully supported by statistical analysis, the distributions of scaling and bias values still offer insights into the nature and direction of performance distortions observed in this study. Specifically, there was a modest tendency to speed up the tapping when transitioning from the globally regular sequence AAAAA to the globally deviant AAAAB (Figure S3A). Conversely, when transitioning from the globally regular AAAAB to the globally deviant AAAAA, a slight slowing of tapping during the Synchronization phase and a modest speeding up and anticipation in tap onset during the Continuation phase were observed (Figures S3A and S5A).

#### 3.2.2 Exp2A

In the auditory experiment, the ANOVA on the two models’ estimates for the scaling component (scaling‐synch: *R* ^2^_marg_ = 0.05, *R* ^2^_cond_ = 0.31; scaling‐cont: *R* ^2^_marg_ = 0.07, *R* ^2^_cond_ = 0.71) showed a main effect of sequence type (scaling‐synch: scaling_A_ = 1, scaling_B_ = 0.999, *F*(1, 78) = 4.70, *p* = 0.033, $\eta_{p}^{2}$ = 0.06; scaling‐cont: scaling_A_ = 0.991, scaling_B_ = 0.979, *F*(1, 78) = 12.23, *p* = 0.0008, $\eta_{p}^{2}$ = 0.13) and a significant interaction between trial type and sequence type only during the Continuation phase (*F*(1, 78) = 12.10, *p* = 0.0008, $\eta_{p}^{2}$ = 0.13; all effects reported in Table S8). For both effects, these results indicated a general slowing of the tapping when participants reproduced the locally deviant sequence AAAAB compared to the locally regular AAAAA (Figures 4B,D and S3B). However, pairwise comparison tests for the Continuation model revealed that this distortion reached statistical significance only when AAAAB was also globally deviant (GlobaReg_A_—GlobDev_B_ = 0.016, *t*(78) = 3.26, *p* = 0.009; GlobDev_B_—GlobReg_B_ = −0.016, *t*(78) = −3.25, *p* = 0.009; GlobDev_A_—GlobDev_B_ = 0.024, *t*(78) = 4.93, *p* <0.0001; all contrasts and means reported in Table S12). The same pattern of results emerged for the two models on the bias component (bias‐synch: *R* ^2^_marg_ = 0.04, *R* ^2^_cond_ = 0.43; bias‐cont: *R* ^2^_marg_ = 0.07, *R* ^2^_cond_ = 0.71). The ANOVA on models’ estimates showed a main effect of sequence type (bias‐synch: bias_A_ = 0.055, bias_B_ = 0.082, *F*(1, 78) = 4.82, *p* = 0.031, $\eta_{p}^{2}$ = 0.06; bias‐cont: bias_A_ = 0.286, bias_B_ = 0.583, *F*(1, 78) = 12.26, *p* = 0.0008, $\eta_{p}^{2}$ = 0.13) and a significant interaction between trial type and sequence type only during the Continuation phase (*F*(1, 78) = 11.15, *p* = 0.0013, $\eta_{p}^{2}$ = 0.12; all effects reported in Table S14). In both cases, these results indicated a significant delay when tapping the locally deviant sequence AAAAB compared to the locally regular AAAAA (Figures S4B,D and S5B). Again, during the Continuation phase, this distortion was statistically greater only when AAAAB was also globally deviant (GlobaReg_A_—GlobDev_B_ = −0.39, *t*(78) = −3.25, *p* = 0.009; GlobDev_B_—GlobReg_B_ = 0.38, *t*(78) = 3.14, *p* = 0.013; GlobDev_A_—GlobDev_B_ = −0.58, *t*(78) = −4.84, *p* <0.0001), as revealed by pairwise comparison tests (all contrasts and means reported in Table S17).

#### 3.2.3 Exp3VC

In the experiment using complex visual sequences, the ANOVA on the two LME models’ estimates for the scaling component (scaling‐synch: *R* ^2^_marg_ = 0.01, *R* ^2^_cond_ = 0.57; scaling‐cont: *R* ^2^_marg_ = 0.01, *R* ^2^_cond_ = 0.87) showed only a main effect of sequence type during the Continuation phase (scaling‐cont: scaling_A_ = 1.022, scaling_B_ = 1.009, *F*(1, 75) = 8.84, *p* = 0.0039, $\eta_{p}^{2}$ = 0.10; all effects reported in Table S9), due to higher scaling values for the locally regular compared to the locally deviant sequence. This difference indicates a general speeding up of the tapping when performing the locally regular CBAAA compared to the locally deviant CBAAB during the Continuation phase (Figures 4C,D and S3C), as reflected by scaling values of the locally regular CBAAA being consistently above baseline (i.e., >1) and higher than those of the locally deviant CBAAB. A similar set of results was observed for the two models on the bias component (bias‐synch: *R* ^2^_marg_ = 0.007, *R* ^2^_cond_ = 0.54; bias‐cont: *R* ^2^_marg_ = 0.009, *R* ^2^_cond_ = 0.88). The ANOVA on models’ estimates revealed, again, only a main effect of sequence type during the Continuation phase (bias‐cont: bias_A_ = −0.632, bias_B_ = −0.254, *F*(1, 75) = 6.84, *p* = 0.011, $\eta_{p}^{2}$ = 0.08; all effects reported in Table S15), with more negative bias values for locally regular compared to locally deviant sequences. This difference reflects a significant tendency to anticipate tapping onsets when performing the locally regular CBAAA compared to the locally deviant CBAAB in the Continuation phase (Figures S4C,D and S5C), as bias values of CBAAA are generally lower than baseline (i.e., <0) and lower than those of CBAAB.

Overall, results from the LME models on Procrustes components showed that the detrimental effects on tapping precision observed in the MSC analysis could be partially explained by specific and quantifiable distortions in the tapping series, rather than being completely attributable to random noise. Specifically, when performing simple visual sequences that are globally deviant (Exp1V), the tapping is characterized either by mild speeding up for the globally and locally deviant AAAAB, or by fluctuations between acceleration/anticipation and deceleration/delay (depending on the task phase) for the globally deviant but locally regular AAAAA. When the same sequences are experienced in the auditory modality (Exp2A), the tapping is characterized by deceleration and delayed onsets for the locally deviant AAAAB. This effect increases during the Continuation phase when AAAAB is also the globally deviant sequence. When stimuli sequences have more complex structures (Exp3VC), the tapping of the locally regular CBAAA tends to be faster and more anticipatory relative to baseline, but only in the Continuation phase, when participants rely on their internal representation of the sequences. Overall bias and scaling distortions account to a larger extent for the detrimental effects found in Exp2A, as revealed by more robust effect sizes compared to the other experiments. It must be noticed, though, that the LME models fitted on scaling and bias components have low *R* ^2^_marginal_ values, meaning that the variability explained by our experimental manipulations is quite limited. While our models’ results are statistically robust, interpretations based on them have to be made cautiously.

## 4 Discussion

Traditional timing models suggest that humans use different mechanisms to process single intervals and temporal patterns [6]. However, they leave largely unclear whether and how humans process all the features that define a temporal pattern, that is, its individual durations and overarching structure. In the current work, we addressed a narrow aspect of this broad question, adopting a paradigm [15] derived from sequence processing studies. Specifically, we investigated how the reproduction of a temporal pattern is affected by different levels of violation of its regularity, namely, a violation within its elements (local deviance) and of its overall structure (global deviance). In three different experiments, we tested how these violations affect reproduction performance and whether their effects depend on the sensory modality (visual or auditory) in which the patterns are experienced and/or on the complexity of their structure.

Overall, results in the frequency‐domain showed a consistent presence of local deviance effects in all three experiments, such that tapping precision was always affected by violations of the internal regularity of the sequence. However, the results also showed different global deviance effects for visual sequences, according to the degree of complexity of their structures. Specifically, when using simple visual sequences (Exp1V), tapping precision dropped when the violation of the sequence was both at the local and global level (i.e., only when going from AAAAA to AAAAB). When using simple auditory sequences (Exp2A), the local deviance effect was spread across conditions, irrespective of the global status of the sequence, and was stronger compared to the effect found in Exp1V. When using complex visual sequences (Exp3VC), instead, both local and global deviance effects were observed. The local deviance effect was independent of the global status of the sequence and the task phase, whereas the global deviance effect was independent of the local status of the sequence but emerged specifically during the Synchronization phase. This last result suggests that the processing of complex visual temporal patterns may require the integration of local elements into a global structure; consequently, violations of the overall structure have a more detrimental effect. By contrast, when reproducing simpler patterns (as in Exp1V and Exp2A), the local regularity appears to carry more weight, and local changes have the greatest detrimental effects on performance. In relation to this, it should be pointed out that the simple sequences employed in Exp1V and Exp2A are isochronous (or nearly isochronous) patterns. Reproducing such patterns may not require the integration of an overall structure, as all elements are identical, but instead may rely on entrainment‐like mechanisms triggered by the patterns’ intrinsic periodicity [35, 36, 37, 38, 39], which is precisely what local violations perturb. This would be consistent with beat‐based accounts of the mechanism underlying the processing of periodic patterns [40] and would also explain the absence of a pure global deviance effect in both experiments. However, in Exp1V, we did not see a generalized effect of local deviance on tapping precision across conditions, as seen in Exp2A and as would be predicted if the pattern's processing would only rely on its periodicity [40].

To directly investigate the role of entrainment‐like mechanisms in modulating the effects of global and local violations in the visual domain, it would be useful to employ a locally deviant sequence in which the length of the last interval is twice the length of the previous four, that is, it violates the sequence local regularity while still maintaining its internal periodicity. In this case, beat‐based accounts would predict that if an entrainment‐like mechanism was exclusively at play, neither local nor global violations would affect the tapping precision, as both sequences possess internal periodicity that can equally engage such mechanism. Similar to the sequences’ internal periodicity, the sequences’ overall tempo might have influenced the local deviance effects we observed in Exp1V and Exp2A. Indeed, several studies have shown how tapping precision is influenced by the rate of the cue sequence [41], also in a modality‐specific way [42], including how far this rate is from each individual's spontaneous motor tempo (SMT) [43]. We cannot rule out that employing faster or slower sequences, or tailoring the sequences’ tempo based on each participant's SMT, would have led to different local deviance effects, for example, smaller effects of local violations when these violations match the SMT.

Finally, it is interesting to note that the benefit of the local regularity on tapping precision was found also with complex sequences (i.e., the local deviance effect in Exp3VC), in which there is no periodicity at all. This result, together with the simultaneous presence of the global deviance effect, suggests that both local elements and overall structure carry equal weight in the precise reproduction of complex temporal patterns. Hence, both levels may need to be encoded and represented in the brain to efficiently process such patterns, at least when they are experienced in the visual domain.

Overall, we observed a greater precision in performance (i.e., higher MSC values) when using auditory sequences, compared to visual ones, even if they had the same structure. This result is consistent with sensorimotor synchronization literature (for extensive reviews, see Refs. [43] and [44]) and the notion that tapping in synchrony with simple auditory rhythms is characterized by higher accuracy and less variability compared to the synchronization with visual rhythms [19, 45, 46]. This higher precision might also explain the stronger local deviance effect size found in Exp2A compared to Exp1V (i.e., between simple auditory and visual rhythms). Indeed, visuo‐motor synchronization is characterized by a higher baseline variability compared to audio‐motor synchronization [25]. This means that any drop in tapping precision due to local violations may not result in strong observable effects. Conversely, local violations of auditory rhythms may produce more disruptive effects on tapping precision, and thus stronger statistical effects, due to the very low baseline variability of audio‐motor synchronization, as observed in Exp2A.

Procrustes analysis results revealed how deviance effects in tapping precision were partially explained by the presence of systematic biases and a rescaling of the patterns. Specifically, in Exp1V, we observed a weak tendency to speed up and anticipate the tapping of the locally and globally deviant sequence AAAAB; whereas in Exp2A, this tendency was observed in the opposite direction (i.e., slowing down and delaying the tapping of all locally deviant sequences). In Exp3VC, we observed a speeding up of the locally regular sequence compared to both the baseline and the locally deviant sequence, but only when the tapping occurred without an external reference. This last finding cannot be unequivocally attributed to our experimental manipulation, as we cannot rule out the possibility that the speeding up might have turned into a slowing down, had we used a different combination of intervals to build our complex sequences.

In all three studies, scaling and bias distortions were mostly observed during the Continuation phase, when the reference stimulus was absent. This task phase‐specificity might be explained by the absence of error correction mechanisms typically present when tapping along with an external rhythm [47, 48]. During the Continuation phase, participants can rely only on an internal representation of the pattern, which might be slightly distorted from the original stimulus and, in turn, give rise to characteristic “drifts” in the tapping frequency spectrum [44, 49]. In general, the presence of scaling and bias components in all experiments suggests that the general structure of the patterns was at least partially conserved, even if reproduced in a distorted version.

Taken together, our results suggest a nuanced picture relative to the brain mechanisms underlying the processing of temporal patterns, in particular the processing of local and global regularities embedded in them. Based on the extensive literature on the local–global paradigm [12], we can speculate that a similar interplay of automatic processing and top‐down modulation may be required in our task. Low‐level, sensory‐driven processing of local temporal regularities may interact with higher‐level temporal predictions, based on a more abstract representation of the pattern's global regularity, to guide pattern reproduction. This may be the case when processing temporal patterns in the visual domain, where top‐down modulations become increasingly relevant as the structural complexity of the pattern increases. In contrast, for highly periodic auditory patterns, such as those used in our Exp2A, such a nuanced interaction is less likely. Based on the extensive literature on sensorimotor synchronization [43] and the underlying neural mechanisms [50], we can reasonably hypothesize a predominant involvement of the cortico–subcortico–cerebellar network that supports audio‐motor entrainment [37]. However, we cannot exclude a more substantial role of top‐down modulations driven by global regularities also for complex auditory patterns. Studies investigating the processing of and synchronization with complex auditory rhythms (e.g., see Ref. [51]) have shown the involvement of cortical areas associated with sequencing, hierarchical ordering, and motor planning. These regions are strong candidates for representing and guiding the reproduction of complex global regularities. We acknowledge the difficulty of extending our results and related speculations on complex patterns to the auditory domain. It is possible that applying the local–global paradigm to complex auditory sequences would yield deviance effects only partially overlapping with those observed in the visual domain. Based on results from Exp2A and Exp3VC, we can hypothesize that both local and global violations would affect the reproduction of complex auditory patterns; however, local violations, consistent with what we observed in Exp2A, may still exert the most detrimental effect.

In addition to the absence of an experiment testing complex auditory sequences, the present study has other limitations. First, unlike the original version, our variant of the local–global paradigm employed multiple repetitions of the sequences within a trial, even when they were globally deviant, in order to align with the structure of the Synchronization‐Continuation task. These repetitions may have diminished the sequences’ global status as “unexpected” and instead amplified local violation effects, which in turn cannot be straightforwardly equated with oddball‐like behavioral signatures, as intended in the original paradigm [15, 52]. Future adaptations of this paradigm to the temporal domain should consider alternative tasks and incorporate nonisochronous patterns, similar to our Exp3VC, to control for potential confounds related to periodicity [4]. Second, our measure of performance precision, MSC, is sensitive to phase shifts and amplitude differences between stimulus and tapping signals [34]. However, it does not provide information about the intrinsic spectral coherence of tapping performance, independent of the stimulus, either within or across trials. To address this complementary question, future work could integrate MSC with a time‐frequency analysis of tapping performances [53]. Finally, to add completeness to the Procrustes analysis, a further specification of the noise component in the tapping signal would exclude the presence of additional overlooked trends.

To conclude, our findings suggest that local regularities within a temporal pattern strongly influence the precision of its reproduction, no matter the complexity of the pattern or the sensory modality in which it is experienced. In relation to our initial question, this result suggests that the processing of a temporal pattern might invariably require the processing of each element it is made of. However, when dealing with visual temporal patterns, the familiarity of the overall structure acquires more relevance in affecting its reproduction, a relevance that increases as the complexity of the pattern increases. This, in turn, suggests an increasing weight given to the overall structure when processing visual patterns. Our results also showed that the effects of local and global violations do not necessarily manifest as a random disruption of the pattern, but can instead appear as specific distortions quantifiable through internal consistency measures of performance. More generally, the present study suggests that processing and reproducing a temporal pattern involves the interplay between its individual constitutive durations and its overall structure. The relative weighting of local and global levels depends on the sensory modality in which the patterns are experienced and, in the case of visual patterns, on their structural complexity. This differential weighing suggests a nuanced, simultaneous involvement of multiple mechanisms, particularly when visual temporal patterns contain multiple layers of nonperiodic regularities. Overall, our findings highlight the need to complement traditional experimental approaches with alternative, multifeature paradigms when investigating temporal pattern processing across different levels of complexity and sensory modalities.

## Author Contributions

D.G.: Conceptualization, software, investigation, formal analysis, data curation, writing – original draft, writing – review and editing, visualization. R.B.: Formal analysis, writing – review and editing. G.F.: Conceptualization, software, formal analysis, writing – review and editing. D.B.: Conceptualization, resources, supervision, funding acquisition, project administration, writing – review and editing.

## Funding

The study was supported by the European Research Council (ERC) under the European Union's Horizon 2020 research and innovation program (grant agreement no. 682117 BIT‐ERC‐2015‐CoG), and the Italian Ministry of University and Research under the call PRIN2022 (project ID: 2022CCPJ3J).

## Conflicts of Interest

The authors declare no conflicts of interest.

## Supporting information




**Supplementary Material**: nyas70173‐sup‐0001‐SuppMatt.docx

## Data Availability

Original scripts and data supporting the findings are available at the following link: https://osf.io/g4385/overview
