# Supplementary material for: Global and Local Deviance Effects in the Processing of Temporal Patterns
Source: Ann N Y Acad Sci. 2025 Dec 24;1556(1):e70173. doi: 10.1111/nyas.70173 (PMC12917930; doi:10.1111/nyas.70173)
Supplement: Supplementary file 1 — Supplementary Material: nyas70173‐sup‐0001‐SuppMatt.docx [file NYAS-1556-0-s001.docx]

**Global and local deviance effects in the processing of temporal patterns**

**SUPPLEMENTARY MATERIAL and FIGURES**


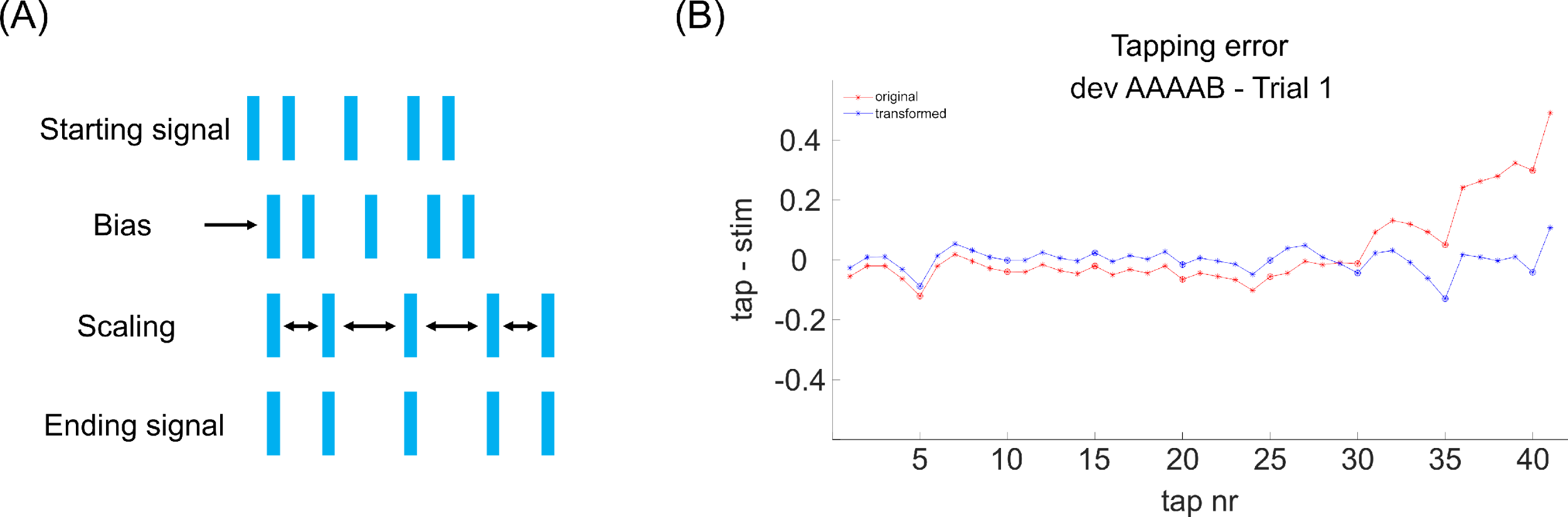


**Figure S1**. Procrustes analysis. (A) Procrustes algorithm applied to time series performs a linear transformation to find the best match between a starting and an ending signal. The best match results in the quantification of a bias and a scaling component, which represent distortions in the ending signal compared to the starting one. The bias corresponds to a translation of events’ onset in a positive (delay) or negative (anticipation) direction, whereas the scaling represents either a stretching or squeezing of events’ onset. (B) Example of a tapping signal before and after applying Procrustes. Tap onsets are plotted as relative differences from stimuli onsets, as a function of tap number. The original signal (in red) shows a systematic negative bias across the whole trial and a scaling component (stretching) that appears in the last 15 taps. Once bias and scaling components are extracted, the transformed signal (in blue) better approximates the stimulus signal and shows a residual variability due to noise.

**Method S1. Procrustes analysis: pre-processing steps.**

In order to pair the length of tapping and stimuli signals, which is a required condition to apply the Procrustes algorithm, the following pre-processing steps were performed for all three experiments (in addition to the initial pre-processing described in the main manuscript at paragraph *Preprocessing*). First, all stimuli and taps onsets detected earlier than 250 ms before the 1^st^ stimulus onset of the Synchronization phase were removed. Of these tapping series, those with less than 31 taps were discarded (<0.05% of the total number of trials) to ensure that all series used for the Procrustes analysis contained enough data from both the Synchronization and Continuation phases. Tapping series were then aligned to stimuli series based on a minimum absolute difference criterion in their timing onsets during the first part of the Synchronization phase (for example, the best alignment of the two series could result as 1^st^ tap - 3^rd^ stimulus, 2^nd^ tap - 4^th^ stimulus, and so on). Gaps between consecutive tapping onsets that exceeded 1.5 times the longest interval between two consecutive stimuli onsets were classified as “missing tap”. The stimulus onset closest to each such gap was excluded from the series. The two resulting aligned series were then matched in length by setting the starting point at the time of the first tap, and defining the endpoint based on the shorter of the two series (tap or stimulus).

**
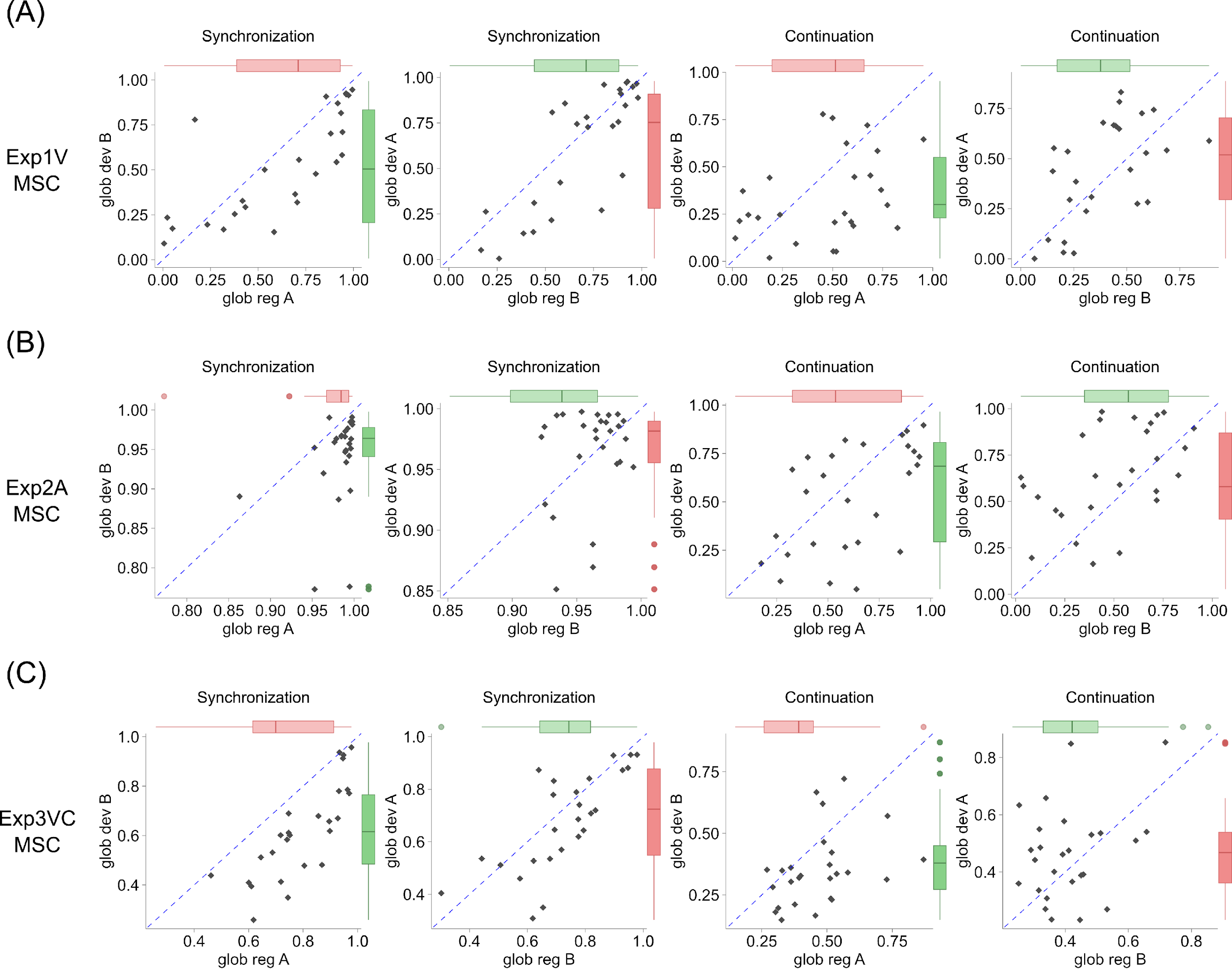
**

**Figure S2**. Magnitude Squared Coherence (MSC) analysis. Each scatter plot shows the distribution of MSC values in each experimental condition (block type) and task phase, for Exp1V (A), Exp2A (B) and Exp3VC (C). Each dot represents the mean MSC value for one participant for the globally regular sequence (x-axis) plotted against the mean MSC value of its respective globally deviant sequence (y-axis), either in the Synchronization or Continuation phase. For example, the plot on the top left shows MSC values for the globally regular AAAAA plotted against the globally deviant AAAAB for the Synchronization phase in Exp1V. The identity line allows to visualize if there is a worsening (dots below the line) or improvement (dots above the line) in mean MSC when transitioning from the globally regular to the globally deviant sequence. In the top left plot of the example it can be noticed that almost all participants show a clear drop in MSC values when performing the globally deviant sequence AAAAB, compared to the globally regular AAAAA. The boxplots along the horizontal (globally regular sequence) and vertical (globally deviant sequence) axes represent each distribution of MSC values at the group level (color-code: light red = globally regular A; green = globally deviant B; light green = globally regular B; red = globally deviant A).

**Magnitude Squared Coherence analysis – ANOVA on Linear Mixed Effects models**

| Exp1V - Type III Anova on LME model estimate | | | | | |  |
| --- | --- | --- | --- | --- | --- | --- |
|  | **Sum Sq** | **NumDF** | **DenDF** | **F value** | **Pr(>F)** | **η^2^_p_ [95% C.I.]** |
| TrialType | 0.200 | 1 | 175 | 6.051 | **0.015** | **0.033 [0.004 1]** |
| SeqType | 0.130 | 1 | 175 | 3.941 | **0.049** | **0.02 [<0.001, 1]** |
| PhaseType | 2.249 | 1 | 175 | 68.018 | **<0.0001** | **0.28 [0.19, 1]** |
| TrialType:SeqType | 0.108 | 1 | 175 | 3.257 | 0.073 | 0.018 [0, 1] |
| TrialType:PhaseType | 0.027 | 1 | 175 | 0.810 | 0.369 | 0.005 [0, 1] |
| SeqType:PhaseType | 0.062 | 1 | 175 | 1.883 | 0.172 | 0.01 [0, 1] |
| TrialType:SeqType:PhaseType | 0.048 | 1 | 175 | 1.465 | 0.228 | 0.008 [0, 1] |

**Table S1**. Type III Anova on Linear Mixed Effects (LME) model estimate for Magnitude Squared Coherence (MSC) values of **Exp1V**.

| Exp2A - Type III Anova on LME model estimate | | | | | |  |
| --- | --- | --- | --- | --- | --- | --- |
|  | **Sum Sq** | **NumDF** | **DenDF** | **F value** | **Pr(>F)** | **η^2^_p_ [95% C.I.]** |
| TrialType | <0.001 | 1 | 182 | 0.001 | 0.982 | < 0.001 [0, 1] |
| SeqType | 0.333 | 1 | 182 | 11.942 | **0.0007** | **0.062 [0.017, 1]** |
| PhaseType | 8.023 | 1 | 182 | 287.758 | **<0.0001** | **0.61 [0.54, 1**] |
| TrialType:SeqType | 0.002 | 1 | 182 | 0.087 | 0.768 | < 0.001 [0, 1] |
| TrialType:PhaseType | 0.051 | 1 | 182 | 1.813 | 0.180 | 0.01 [0, 1] |
| SeqType:PhaseType | 0.114 | 1 | 182 | 4.099 | **0.044** | **0.02 [<0.001, 1]** |
| TrialType:SeqType:PhaseType | 0.005 | 1 | 182 | 0.169 | 0.682 | < 0.001 [0, 1] |

**Table S2**. Type III Anova on Linear Mixed Effects LME model estimate for MSC values of **Exp2A**.

| Exp3VC - Type III Anova on LME model estimate | | | | | |  |
| --- | --- | --- | --- | --- | --- | --- |
|  | **Sum Sq** | **NumDF** | **DenDF** | **F value** | **Pr(>F)** | **η^2^_p_ [95% C.I.]** |
| TrialType | 0.271 | 1 | 175 | 20.794 | **<0.0001** | **0.106 [0.045, 1]** |
| SeqType | 0.292 | 1 | 175 | 22.413 | **<0.0001** | **0.114 [0.05, 1]** |
| PhaseType | 4.051 | 1 | 175 | 310.661 | **<0.0001** | **0.64 [0.57, 1]** |
| TrialType:SeqType | 0.007 | 1 | 175 | 0.550 | 0.459 | 0.003 [0, 1] |
| TrialType:PhaseType | 0.092 | 1 | 175 | 7.045 | **0.0087** | **0.04 [0.006, 1]** |
| SeqType:PhaseType | 0.005 | 1 | 175 | 0.387 | 0.534 | 0.002 [0,1] |
| TrialType:SeqType:PhaseType | 0.013 | 1 | 175 | 1.032 | 0.311 | 0.006 [0,1] |

**Table S3**. Type III Anova on Linear Mixed Effects LME model estimate for MSC values of **Exp3VC**.

**Magnitude Squared Coherence analysis – Pairwise comparison tests**

| contrast | estimate | SE | df | t.ratio | p.value |
| --- | --- | --- | --- | --- | --- |
| Dev A - Reg A | -0.017 | 0.036 | 175 | -0.463 | 0.967 |
| Dev A - Dev B | 0.096 | 0.036 | 175 | 2.68 | **0.040** |
| Dev A - Reg B | -0.012 | 0.036 | 175 | -0.336 | 0.987 |
| Reg A - Dev B | 0.112 | 0.036 | 175 | 3.143 | **0.010** |
| Reg A - Reg B | 0.005 | 0.036 | 175 | 0.128 | 0.999 |
| Dev B - Reg B | -0.108 | 0.036 | 175 | -3.015 | **0.015** |

**Table S4**. Pairwise comparison tests on MSC values (interaction **Trial type x Sequence type**) of **Exp1V**.

| contrast | estimate | SE | df | t.ratio | p.value |
| --- | --- | --- | --- | --- | --- |
| A Cont - B Cont | 0.125 | 0.032 | 182 | 3.875 | **0.0008** |
| A Cont - A Sync | -0.339 | 0.032 | 182 | -10.563 | <0.0001 |
| A Cont - B Sync | -0.307 | 0.032 | 182 | -9.551 | <0.0001 |
| B Cont - A Sync | -0.464 | 0.032 | 182 | -14.439 | <0.0001 |
| B Cont - B Sync | -0.431 | 0.032 | 182 | -13.427 | <0.0001 |
| A Sync - B Sync | 0.033 | 0.032 | 182 | 1.012 | 0.743 |

**Table S5**. Pairwise comparison tests on MSC values (interaction **Sequence type x Task Phase**) of **Exp2A**. Comparisons of interest are highlighted in bold in the column “contrast”.

| contrast | estimate | SE | Df | t.ratio | p.value |
| --- | --- | --- | --- | --- | --- |
| Dev Cont - Reg Cont | -0.030 | 0.022 | 175 | -1.348 | 0.534 |
| Dev Cont - Dev Sync | -0.237 | 0.022 | 175 | -10.586 | 0 |
| Dev Cont - Reg Sync | -0.351 | 0.022 | 175 | -15.688 | 0 |
| Reg Cont - Dev Sync | -0.207 | 0.022 | 175 | -9.239 | 0 |
| Reg Cont - Reg Sync | -0.321 | 0.022 | 175 | -14.340 | 0 |
| Dev Sync - Reg Sync | -0.114 | 0.022 | 175 | -5.101 | **<0.0001** |

**Table S6**. Pairwise comparison tests on MSC values (interaction **Trial type x Task Phase**) of **Exp3VC**. Comparisons of interest are highlighted in bold in the column “contrast”.

**
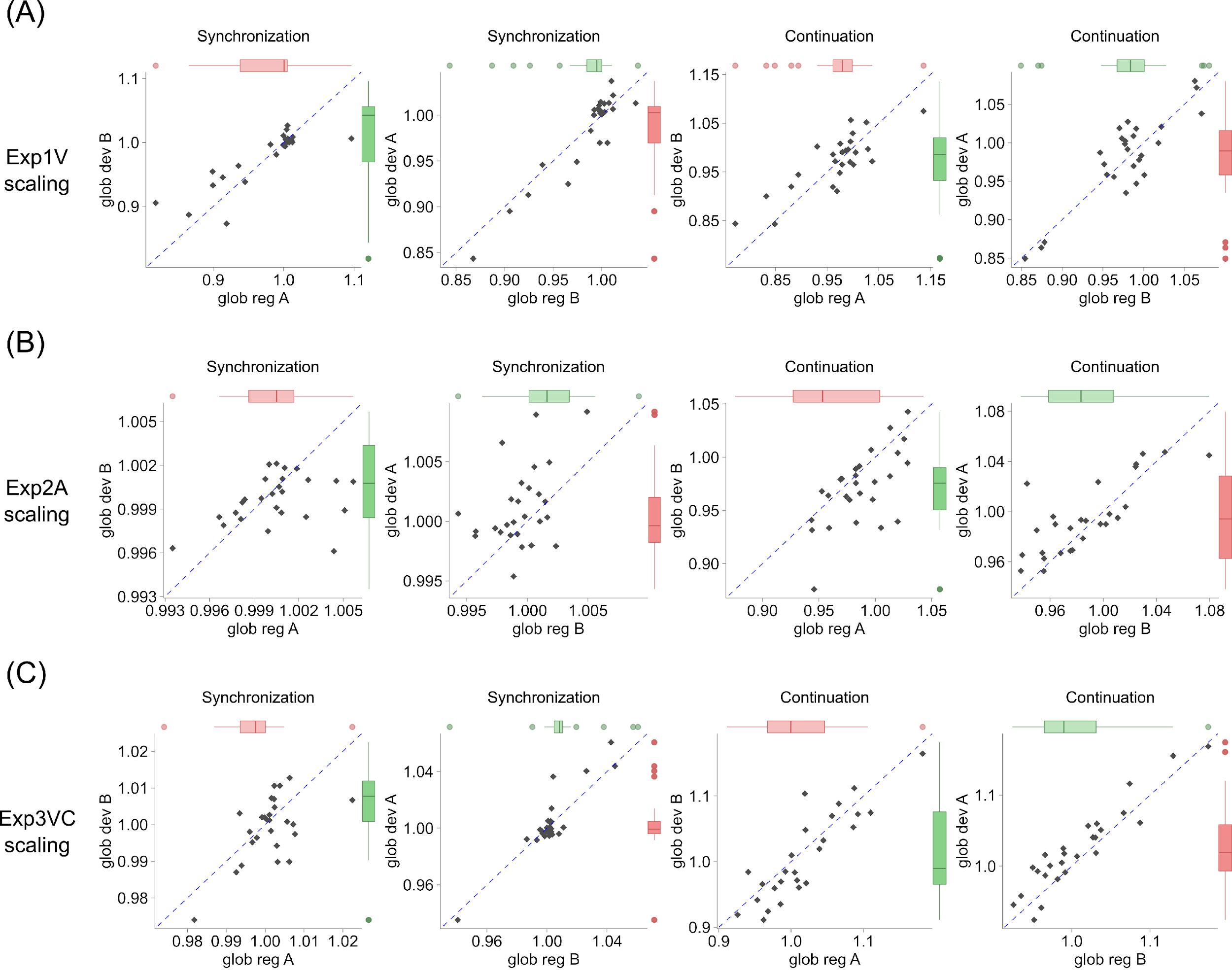
**

**Figure S3**. Procrustes analysis: scaling component. Each scatter plot shows the distribution of scaling values in each experimental condition (block type) and task phase, for Exp1V (A), Exp2A (B) and Exp3VC (C). Each dot represents the mean scaling value for one participant for the globally regular sequence (x-axis) plotted against the mean scaling value of its respective globally deviant sequence (y-axis), either in the Synchronization or Continuation phase. The identity line allows to visualize if there is a slowing down (dots below the line) or speeding up (dots above the line) of the tapping when transitioning from the globally regular to the globally deviant sequence. For example, the plot on the bottom right (Exp3VC - Continuation) shows a speeding up of the tapping when performing the globally deviant sequence CBAAA compared to the globally regular CBAAB. The boxplots along the horizontal (globally regular sequence) and vertical (globally deviant sequence) axes represent each distribution of scaling values at the group level (color-code: light red = globally regular A; green = globally deviant B; light green = globally regular B; red = globally deviant A).

**Procrustes analysis – ANOVA on Linear Mixed Effects Models on Scaling**

| Exp1V - Type III Anova on LME model estimate | | | | | |  |
| --- | --- | --- | --- | --- | --- | --- |
| *SYNCHRONIZATION* |  |  |  |  |  |  |
|  | **Sum Sq** | **NumDF** | **DenDF** | **F value** | **Pr(>F)** | **η^2^_p_ [95% C.I.]** |
| TrialType | 0.00002 | 1 | 75 | 0.056 | 0.814 | < 0.001 [0, 1] |
| SeqType | 0.0003 | 1 | 75 | 0.881 | 0.351 | 0.012 [0, 1] |
| TrialType:SeqType | 0.0015 | 1 | 75 | 4.16 | **0.045** | **0.05 [<0.001, 1]** |
|  | | | | | |  |
| *CONTINUATION* |  |  |  |  |  |  |
|  | **Sum Sq** | **NumDF** | **DenDF** | **F value** | **Pr(>F)** | **η^2^_p_ [95% C.I.]** |
| TrialType | 0.0004 | 1 | 75 | 0.663 | 0.418 | 0.009 [0,1] |
| SeqType | 0.00006 | 1 | 75 | 0.104 | 0.748 | 0.001 [0,1] |
| TrialType:SeqType | 0.003 | 1 | 75 | 5.458 | **0.022** | **0.068 [0.005,1]** |

**Table S7**. Type III Anova on the two Linear Mixed Effects (LME) models’ estimates for the scaling component from the Synchronization and Continuation phases of **Exp1V**.

| Exp2A - Type III Anova on LME model estimate | | | | | |  |
| --- | --- | --- | --- | --- | --- | --- |
| *SYNCHRONIZATION* |  |  |  |  |  |  |
|  | **Sum Sq** | **NumDF** | **DenDF** | **F value** | **Pr(>F)** | **η^2^_p_ [95% C.I.]** |
| TrialType | 0.000001 | 1 | 78 | 0.050 | 0.823 | < 0.001 [0, 1] |
| SeqType | 0.00009 | 1 | 78 | 4.701 | **0.033** | **0.057 [0.002, 1]** |
| TrialType:SeqType | 0.00005 | 1 | 78 | 2.473 | 0.120 | 0.03 [0, 1] |
|  | | | | | |  |
| *CONTINUATION* |  |  |  |  |  |  |
|  | **Sum Sq** | **NumDF** | **DenDF** | **F value** | **Pr(>F)** | **η^2^_p_ [95% C.I.]** |
| TrialType | 0.0004 | 1 | 78 | 1.244 | 0.268 | 0.016 [0,1] |
| SeqType | 0.0038 | 1 | 78 | 12.228 | **0.0008** | **0.135 [0.04, 1]** |
| TrialType:SeqType | 0.0038 | 1 | 78 | 12.101 | **0.0008** | **0.134 [0.04, 1]** |

**Table S8**. Type III Anova on the two LME models’ estimates for the scaling component from the Synchronization and Continuation phases of **Exp2A**.

| Exp3VC - Type III Anova on LME model estimate | | | | | |  |
| --- | --- | --- | --- | --- | --- | --- |
| *SYNCHRONIZATION* |  |  |  |  |  |  |
|  | **Sum Sq** | **NumDF** | **DenDF** | **F value** | **Pr(>F)** | **η^2^_p_ [95% C.I.]** |
| TrialType | 0.000004 | 1 | 75 | 0.040 | 0.842 | < 0.001 [0, 1] |
| SeqType | 0.00005 | 1 | 75 | 0.507 | 0.479 | 0.007 [0, 1] |
| TrialType:SeqType | 0.00021 | 1 | 75 | 1.996 | 0.162 | 0.026 [0, 1] |
|  | | | | | |  |
| *CONTINUATION* |  |  |  |  |  |  |
|  | **Sum Sq** | **NumDF** | **DenDF** | **F value** | **Pr(>F)** | **η^2^_p_ [95% C.I.]** |
| TrialType | 0.0002 | 1 | 75 | 0.296 | 0.588 | 0.004 [0,1] |
| SeqType | 0.0043 | 1 | 75 | 8.844 | **0.0039** | **0.105 [0.02, 1]** |
| TrialType:SeqType | 0.0002 | 1 | 75 | 0.486 | 0.488 | 0.006 [0, 1] |

**Table S9**. Type III Anova on the two LME models’ estimates for the scaling component from the Synchronization and Continuation phases of **Exp3VC**.

**Procrustes analysis – Pairwise comparison tests on Scaling**

| contrast | estimate | SE | df | t.ratio | p.value |
| --- | --- | --- | --- | --- | --- |
| Dev A - Reg A | 0.009 | 0.005 | 75 | 1.609 | 0.380 |
| Dev A - Dev B | 0.004 | 0.005 | 75 | 0.779 | 0.864 |
| Dev A - Reg B | -0.003 | 0.005 | 75 | -0.497 | 0.960 |
| Reg A - Dev B | -0.004 | 0.005 | 75 | -0.830 | 0.840 |
| Reg A - Reg B | -0.011 | 0.005 | 75 | -2.106 | 0.161 |
| Dev B - Reg B | -0.007 | 0.005 | 75 | -1.275 | 0.581 |
|  |  |  |  |  |  |
| estim. means |  |  |  |  |  |
| TrialType | **SeqType** | **emmean** | **SE** | **lower.CL** | **upper.CL** |
| Dev | **A** | 0.983 | 0.009 | 0.964 | 1.002 |
| Reg | **A** | 0.974 | 0.009 | 0.956 | 0.993 |
| Dev | **B** | 0.979 | 0.009 | 0.960 | 0.997 |
| Reg | **B** | 0.986 | 0.009 | 0.967 | 1.004 |

**Table S10**. Pairwise comparison tests and marginal means (interaction **Trial type x Sequence type**) on scaling values for the **Synchronization** phase of **Exp1V.**

| contrast | estimate | SE | df | t.ratio | p.value |
| --- | --- | --- | --- | --- | --- |
| Dev A - Reg A | 0.015 | 0.007 | 75 | 2.228 | 0.125 |
| Dev A - Dev B | 0.010 | 0.007 | 75 | 1.424 | 0.489 |
| Dev A - Reg B | 0.002 | 0.007 | 75 | 0.348 | 0.985 |
| Reg A - Dev B | -0.005 | 0.007 | 75 | -0.803 | 0.853 |
| Reg A - Reg B | -0.013 | 0.007 | 75 | -1.88 | 0.245 |
| Dev B - Reg B | -0.007 | 0.007 | 75 | -1.076 | 0.705 |
|  |  |  |  |  |  |
| estim. means |  |  |  |  |  |
| TrialType | **SeqType** | **emmean** | **SE** | **lower.CL** | **upper.CL** |
| Dev | **A** | 0.981 | 0.012 | 0.957 | 1.005 |
| Reg | **A** | 0.966 | 0.012 | 0.942 | 0.990 |
| Dev | **B** | 0.972 | 0.012 | 0.948 | 0.996 |
| Reg | **B** | 0.979 | 0.012 | 0.955 | 1.003 |

**Table S11**. Pairwise comparison tests and marginal means (interaction **Trial type x Sequence type**) on scaling values for the **Continuation** phase of **Exp1V.**

| contrast | estimate | SE | df | t.ratio | p.value |
| --- | --- | --- | --- | --- | --- |
| Dev A - Reg A | 0.008 | 0.005 | 78 | 1.671 | 0.346 |
| Dev A - Dev B | 0.024 | 0.005 | 78 | 4.932 | **<0.0001** |
| Dev A - Reg B | 0.008 | 0.005 | 78 | 1.684 | 0.339 |
| Reg A - Dev B | 0.016 | 0.005 | 78 | 3.261 | **0.009** |
| Reg A - Reg B | 0.00006 | 0.005 | 78 | 0.013 | 0.999 |
| Dev B - Reg B | -0.016 | 0.005 | 78 | -3.248 | **0.009** |
|  |  |  |  |  |  |
| estim. means |  |  |  |  |  |
| TrialType | **SeqType** | **emmean** | **SE** | **lower.CL** | **upper.CL** |
| Dev | **A** | 0.995 | 0.006 | 0.983 | 1.007 |
| Reg | **A** | 0.987 | 0.006 | 0.975 | 0.999 |
| Dev | **B** | 0.971 | 0.006 | 0.959 | 0.984 |
| Reg | **B** | 0.987 | 0.006 | 0.975 | 0.999 |

**Table S12**. Pairwise comparison tests and marginal means (interaction **Trial type x Sequence type**) on scaling values for the **Continuation** phase of **Exp2A.**


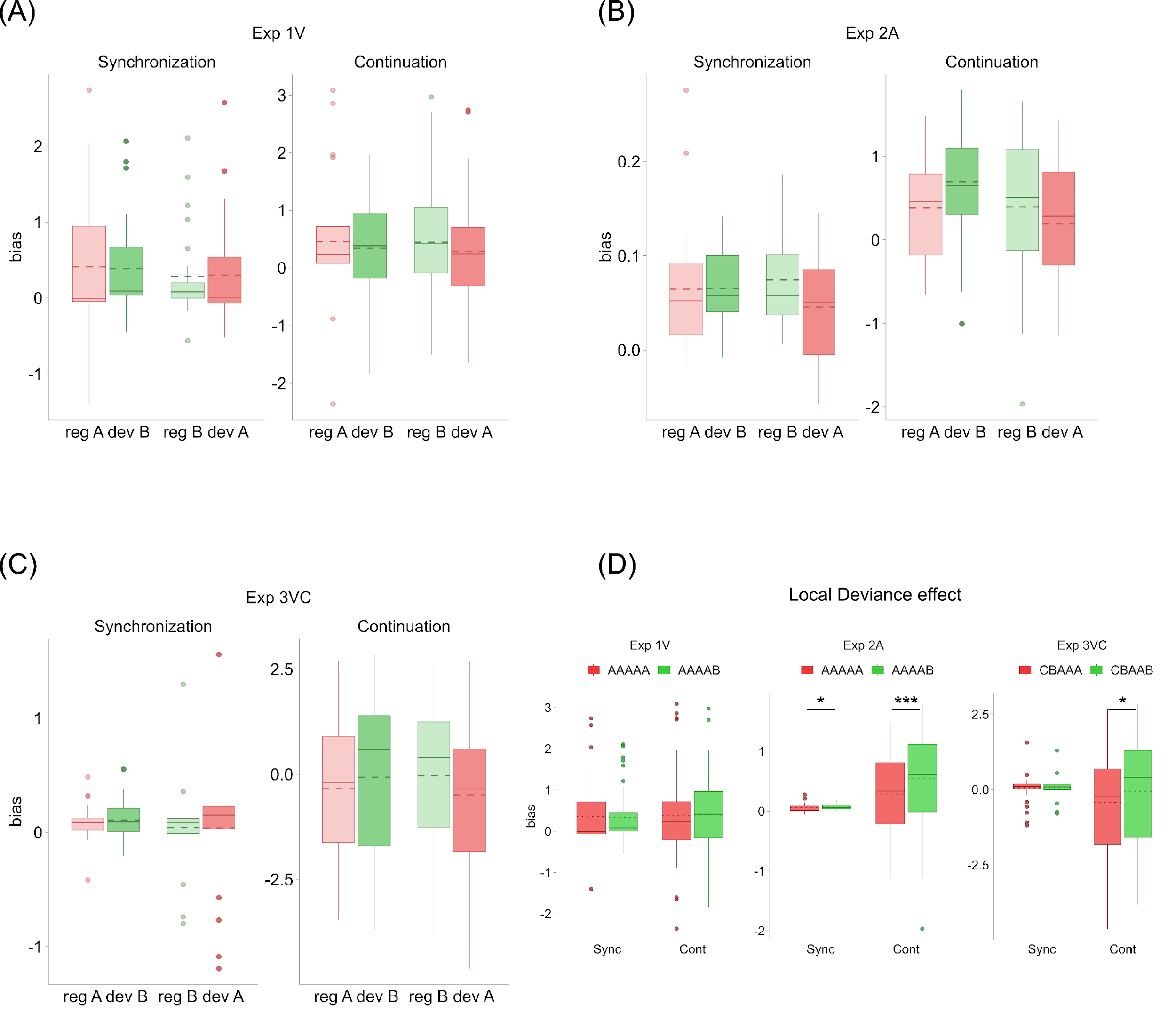


**Figure S4**. Procrustes analysis: bias component. (A) Distribution of bias values for Exp1V grouped by task phase, trial type and sequence type (color-code: light red = globally regular A; green = globally deviant B; light green = globally regular B; red = globally deviant A). In Exp1V only mild differences in bias values were observed between each globally regular sequence and its respective globally deviant sequence, but none of these tendencies was statistically strong. (B) Distribution of bias values for Exp2A grouped and color-coded as in (A). Statistical analysis revealed a general local deviance effect, shown in (D). (C) Distribution of bias values for Exp3VC grouped and color-coded as in (A). Also for complex sequences a statistically significant effect of local deviance was observed. (D) Local deviance effect on bias values for Exp1V (left), Exp2A (center) and Exp3VC (right). In Exp2A and Exp3VC bias values differed significantly between locally regular and locally deviant sequences, whereas in Exp1V they did not. However, bias differences in Exp2A and Exp3VC reflect opposite tapping behaviors: in Exp2A the tap onsets of the locally deviant AAAAB were delayed compared to the baseline (positive bias values) and to the locally regular AAAAA, whereas in Exp3VC the tap onsets of the locally regular CBAAA were anticipated compared to the baseline (negative bias values) and to the locally deviant CBAAB. Boxplots’ borders represent interquartile range, solid and dotted lines represent median and mean bias values, respectively. **p* < 0.05; ****p* < 0.001.

**
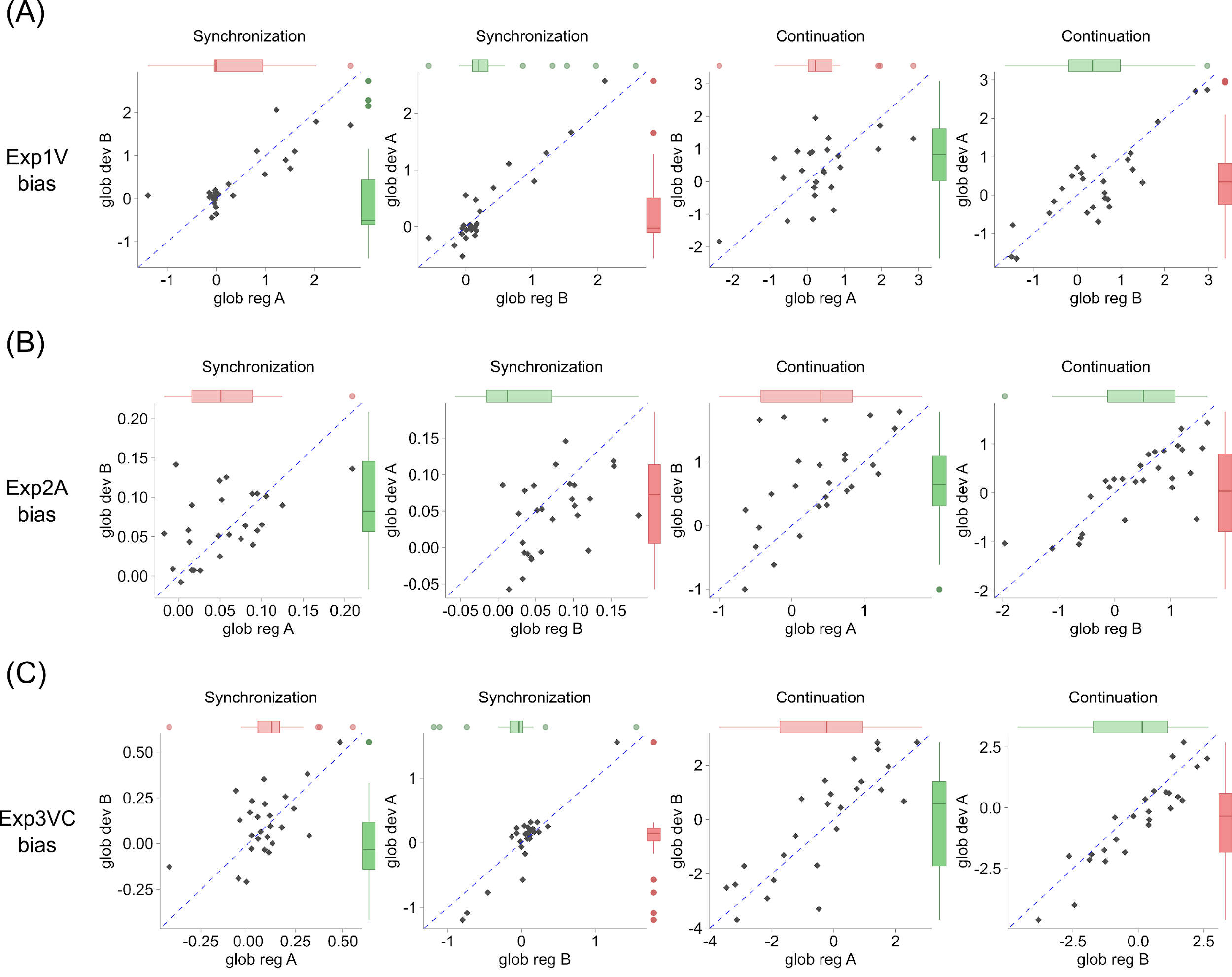
**

**Figure S5**. Procrustes analysis: bias component. Each scatter plot shows the distribution of bias values in each experimental condition (block type) and task phase, for Exp1V (A), Exp2A (B) and Exp3VC (C). Each dot represents the mean bias value for one participant for the globally regular sequence (x-axis) plotted against the mean bias value of its respective globally deviant sequence (y-axis), either in the Synchronization or Continuation phase. The identity line allows to visualize if there is an anticipation (dots below the line) or a delay (dots above the line) in tapping onset when transitioning from the globally regular to the globally deviant sequence. For example, the plot on the top right (Exp1V - Continuation) reveals that a portion of participants tended to slightly anticipate tapping onsets when performing the globally deviant sequence AAAAA compared to the globally regular AAAAB. The boxplots along the horizontal (globally regular sequence) and vertical (globally deviant sequence) axes represent each distribution of scaling values at the group level (color-code: light red = globally regular A; green = globally deviant B; light green = globally regular B; red = globally deviant A).

**Procrustes analysis – ANOVA on Linear Mixed Effects Models on Bias**

| Exp1V - Type III Anova on LME model estimate | | | | | |  |
| --- | --- | --- | --- | --- | --- | --- |
| *SYNCHRONIZATION* |  |  |  |  |  |  |
|  | **Sum Sq** | **NumDF** | **DenDF** | **F value** | **Pr(>F)** | **η^2^_p_ [95% C.I.]** |
| TrialType | 0.001 | 1 | 75 | 0.014 | 0.907 | < 0.001 [0, 1] |
| SeqType | 0.010 | 1 | 75 | 0.117 | 0.734 | 0.002 [0,1] |
| TrialType:SeqType | 0.310 | 1 | 75 | 3.593 | 0.062 | 0.045 [0,1] |
|  | | | | | |  |
| *CONTINUATION* |  |  |  |  |  |  |
|  | **Sum Sq** | **NumDF** | **DenDF** | **F value** | **Pr(>F)** | **η^2^_p_ [95% C.I.]** |
| TrialType | 0.151 | 1 | 75 | 0.56 | 0.456 | 0.007 [0,1] |
| SeqType | 0.163 | 1 | 75 | 0.603 | 0.434 | 0.008 [0,1] |
| TrialType:SeqType | 1.278 | 1 | 75 | 4.74 | **0.033** | **0.06 [0.003,1]** |

**Table S13**. Type III Anova on the two Linear Mixed Effects (LME) models’ estimates for the bias component from the Synchronization and Continuation phases of **Exp1V**.

| Exp2A - Type III Anova on LME model estimate | | | | | |  |
| --- | --- | --- | --- | --- | --- | --- |
| *SYNCHRONIZATION* |  |  |  |  |  |  |
|  | **Sum Sq** | **NumDF** | **DenDF** | **F value** | **Pr(>F)** | **η^2^_p_ [95% C.I.]** |
| TrialType | 0.00009 | 1 | 78 | 0.024 | 0.877 | < 0.001 [0,1] |
| SeqType | 0.019 | 1 | 78 | 4.823 | **0.031** | **0.06 [0.003,1]** |
| TrialType:SeqType | 0.008 | 1 | 78 | 1.956 | 0.166 | 0.024 [0, 1] |
|  | | | | | |  |
| *CONTINUATION* |  |  |  |  |  |  |
|  | **Sum Sq** | **NumDF** | **DenDF** | **F value** | **Pr(>F)** | **η^2^_p_ [95% C.I.]** |
| TrialType | 0.235 | 1 | 78 | 1.208 | 0.275 | 0.015 [0,1] |
| SeqType | 2.390 | 1 | 78 | 12.263 | **0.0008** | **0.135 [0.04,1]** |
| TrialType:SeqType | 2.173 | 1 | 78 | 11.150 | **0.0013** | **0.125 [0.03,1]** |

**Table S14**. Type III Anova on the two LME models’ estimates for the bias component from the Synchronization and Continuation phases of **Exp2A**.

| Exp3VC - Type III Anova on LME model estimate | | | | | |  |
| --- | --- | --- | --- | --- | --- | --- |
| *SYNCHRONIZATION* |  |  |  |  |  |  |
|  | **Sum Sq** | **NumDF** | **DenDF** | **F value** | **Pr(>F)** | **η^2^_p_ [95% C.I.]** |
| TrialType | 0.003 | 1 | 75 | 0.05 | 0.824 | < 0.001 [0,1] |
| SeqType | 0.005 | 1 | 75 | 0.09 | 0.765 | 0.001 [0,1] |
| TrialType:SeqType | 0.080 | 1 | 75 | 1.51 | 0.224 | 0.02 [0,1] |
|  | | | | | |  |
| *CONTINUATION* |  |  |  |  |  |  |
|  | **Sum Sq** | **NumDF** | **DenDF** | **F value** | **Pr(>F)** | **η^2^_p_ [95% C.I.]** |
| TrialType | 0.137 | 1 | 75 | 0.253 | 0.616 | 0.003 [0,1] |
| SeqType | 3.716 | 1 | 75 | 6.843 | **0.011** | **0.08 [0.01,1]** |
| TrialType:SeqType | 0.028 | 1 | 75 | 0.051 | 0.822 | < 0.001 [0,1] |

**Table S15**. Type III Anova on the two LME models’ estimates for the bias component from the Synchronization and Continuation phases of **Exp3VC**.

**Procrustes analysis – Pairwise comparison tests on Bias**

| contrast | estimate | SE | df | t.ratio | p.value |
| --- | --- | --- | --- | --- | --- |
| Dev A - Reg A | -0.298 | 0.144 | 75 | -2.069 | 0.173 |
| Dev A - Dev B | -0.301 | 0.144 | 75 | -2.089 | 0.166 |
| Dev A - Reg B | -0.155 | 0.144 | 75 | -1.078 | 0.704 |
| Reg A - Dev B | -0.003 | 0.144 | 75 | -0.020 | 0.999 |
| Reg A - Reg B | 0.147 | 0.144 | 75 | 0.991 | 0.755 |
| Dev B - Reg B | 0.146 | 0.144 | 75 | 1.010 | 0.744 |
|  |  |  |  |  |  |
| estim. means |  |  |  |  |  |
| TrialType | **SeqType** | **emmean** | **SE** | **lower.CL** | **upper.CL** |
| Dev | **A** | 0.290 | 0.234 | -0.186 | 0.766 |
| Reg | **A** | 0.588 | 0.234 | 0.112 | 1.064 |
| Dev | **B** | 0.591 | 0.234 | 0.115 | 1.067 |
| Reg | **B** | 0.446 | 0.234 | -0.031 | 0.922 |

**Table S16**. Pairwise comparison tests and marginal means (interaction **Trial type x Sequence type**) on bias values for the **Continuation** phase of **Exp1V.**

| contrast | estimate | SE | df | t.ratio | p.value |
| --- | --- | --- | --- | --- | --- |
| Dev A - Reg A | -0.190 | 0.120 | 78 | -1.584 | 0.394 |
| Dev A - Dev B | -0.581 | 0.120 | 78 | -4.837 | **<0.0001** |
| Dev A - Reg B | -0.204 | 0.120 | 78 | -1.699 | 0.331 |
| Reg A - Dev B | -0.391 | 0.120 | 78 | -3.253 | **0.009** |
| Reg A - Reg B | -0.014 | 0.120 | 78 | -0.115 | 0.999 |
| Dev B - Reg B | 0.377 | 0.120 | 78 | 3.138 | **0.013** |
|  |  |  |  |  |  |
| estim. means |  |  |  |  |  |
| TrialType | **SeqType** | **emmean** | **SE** | **lower.CL** | **upper.CL** |
| Dev | **A** | 0.191 | 0.151 | -0.114 | 0.495 |
| Reg | **A** | 0.381 | 0.151 | 0.077 | 0.685 |
| Dev | **B** | 0.772 | 0.151 | 0.468 | 1.076 |
| Reg | **B** | 0.395 | 0.151 | 0.091 | 0.699 |

**Table S17**. Pairwise comparison tests and marginal means (interaction **Trial type x Sequence type**) on bias values for the **Continuation** phase of **Exp2A**.
